# Effectiveness of training methods for delivery of evidence-based psychotherapies: a systematic review

**DOI:** 10.1186/s13012-020-00998-w

**Published:** 2020-05-27

**Authors:** Helen Valenstein-Mah, Nancy Greer, Lauren McKenzie, Lucas Hansen, Thad Q. Strom, Shannon Wiltsey Stirman, Timothy J. Wilt, Shannon M. Kehle-Forbes

**Affiliations:** 1 Department of Psychiatry and Behavioral Sciences, University of Minnesota Medical School, 2450 Riverside Avenue, F282/2A West, Minneapolis, MN 55454 USA; 2 Center for Care Delivery and Outcomes Research, Minneapolis VA Health Care System, Minneapolis, MN USA; 3 Minneapolis VA Health Care System, Minneapolis, MN USA; 4 University of St. Thomas, Minneapolis, MN USA; 5 Oscar G. Johnson VA Medical Center, Iron Mountain, MI USA; 6 National Center for PTSD Dissemination and Training Division, VA Palo Alto Healthcare System, Palo Alto, CA USA; 7 Stanford University, Palo Alto, CA USA; 8 Department of Medicine, University of Minnesota School of Medicine, Minneapolis, MN USA; 9 National Center for PTSD Women’s Health Sciences Division, VA Boston Healthcare System, Boston, MA USA

**Keywords:** Evidence-based psychotherapy, Provider training, Training methods, Adherence, Competence, Fidelity

## Abstract

**Background:**

Extensive efforts have been made to train mental health providers in evidence-based psychotherapies (EBPs); there is increasing attention focused on the methods through which providers are trained to deliver EBPs. Evaluating EBP training methods is an important step in determining which methods are most effective in increasing provider skill and improving client outcomes.

**Methods:**

We searched MEDLINE (Ovid) and PsycINFO for randomized controlled trials published from 1990 through June 2019 that evaluated EBP training methods to determine the effectiveness of EBP training modalities on implementation (provider and cost) and client outcomes. Eligible studies (*N* = 28) were evaluated for risk of bias, and the overall strength of evidence was assessed for each outcome. Data was extracted by a single investigator and confirmed by a second; risk of bias and strength of evidence were independently rated by two investigators and determined by consensus.

**Results:**

Overall, EBP training improved short-term provider satisfaction, EBP knowledge, and adherence compared to no training or self-study of training materials (low to moderate strength of evidence). Training in an EBP did not increase treatment adoption compared to no training or self-study. No specific active EBP training modality was found to consistently increase provider EBP knowledge, skill acquisition/adherence, competence, adoption, or satisfaction compared to another active training modality. Findings were mixed regarding the additive benefit of post-training consultation on these outcomes. No studies evaluated changes in provider outcomes with regards to training costs and few studies reported on client outcomes.

**Limitations:**

The majority of included studies had a moderate risk of bias and strength of evidence for the outcomes of interest was generally low or insufficient. Few studies reported effect sizes. The ability to identify the most effective EBP training methods was limited by low strength of evidence for the outcomes of interest and substantial heterogeneity among studies.

**Conclusions:**

EBP training may have increased short-term provider satisfaction, EBP knowledge, and adherence though not adoption. Evidence was insufficient on training costs and client outcomes. Future research is needed on EBP training methods, implementation, sustainability, client outcomes, and costs to ensure efforts to train providers in EBPs are effective, efficient, and durable.

**Trial registration:**

The protocol for this review is registered in PROSPERO (CRD42018093381).

Contributions to the literature

- Training in evidence-based psychotherapies (EBPs) probably increased short-term provider satisfaction, treatment knowledge, and adherence. Training in an EBP may not have increased EBP adoption compared to no training or self-study.
- No active training modality demonstrated clear superiority over another for provider implementation outcomes. Findings were mixed regarding the additive benefit of post-training consultation on these outcomes. While data was limited, costs of training methods were highest with in-person training and lowest with online training.
- Strength of evidence for outcomes was generally low or insufficient. Future research should focus on training costs and client outcomes as well as sustainability of training effects.

## Background

Extensive efforts have been made to train mental health providers in evidence-based psychotherapies (EBPs)—treatments that have been empirically evaluated and have demonstrated effectiveness in controlled research studies [1]. Substantial financial and organizational resources have been put toward disseminating and implementing EBPs through nationally funded agencies such as the Substance Abuse and Mental Health Services Administration (SAMHSA) and the National Child Traumatic Stress Network (NCTSN), state-funded initiatives, and public healthcare systems such as the Veterans Health Administration (VHA) [2, 3]. In community settings, legislators have passed policies mandating that evidence-based practices be implemented in public mental healthcare [4, 5]. One institutional exemplar in training providers in EBPs is the VHA, the largest integrated healthcare system in the USA. VHA has developed a handbook with expectations and procedures for implementing EBPs at Veterans Affairs (VA) institutions [6] and has trained over 11,600 mental health staff in at least one EBP since 2007 [7]. Taken together, these diverse efforts to implement EBPs highlight both the need, and rapidly growing demand, for access to effective mental health treatment.

Alongside efforts to implement EBPs, there is increasing attention focused on the methods through which providers are trained to deliver EBPs. Evaluating EBP training and determining “evidence-based training” methods is of considerable importance [8]. While earlier research is mixed [9], recent evidence suggests that the fidelity with which EBPs are delivered affects client outcomes, emphasizing the need for well-trained providers [10, 11]. Conversely, ineffective training that does not enhance providers’ EBP skill and competency could result in poorer health outcomes than those reported in controlled trials demonstrating EBP efficacy.

**Fig. 1:**
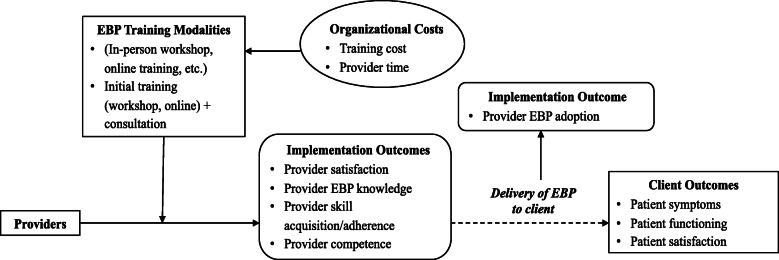
Analytic framework for evaluation of evidence-based psychotherapy (EBP) training methods based on Proctor et al. (2011) conceptual framework for implementation outcomes

The effectiveness of EBP training may be evaluated in the context of different implementation outcomes. Proctor and colleagues (2011) identify three distinct, although interrelated, outcome domains: implementation, service, and client outcomes [12]. Provider-level outcomes typically targeted through EBP training can be considered under the umbrella of implementation outcomes and include (a) *satisfaction* (acceptability of the training to providers), (b) *adoption* (use of EBP by a provider post-training), (c) *knowledge* (understanding of EBP principles and techniques), (d) *skill acquisition* or *adherence* (ability to employ core techniques/interventions of an EBP), (e) *competence* (skill with which EBP techniques are delivered), and (f) *fidelity* (composite of adherence and competence to EBP) [9, 13]. Additional outcomes at the implementation-level include training costs such as financial resources and provider time, including lost productivity and client care hours due to time spent in training activities. Service outcomes, derived from the Institute of Medicine’s quality improvement aims, were not directly examined in this review. Finally, client outcomes include symptom reduction, functional improvement, and treatment satisfaction (see Fig. 1 for conceptual model).

The use of different implementation strategies [14] may lead to more or less successful implementation outcomes. Some EBP training methods, such as self-directed study of treatment materials and in-person workshops without follow-up, do not increase provider skill acquisition [15, 16]. More intensive training methods may lead to meaningful gains in provider skill in delivering an EBP. For example, an earlier review found evidence that workshops with additional follow-up and “multi-component” trainings that included observation, feedback, consultation, and coaching increased skill acquisition [15]. However, it remains unclear if more rigorous training methods that may improve provider outcomes also improve client outcomes. Additionally, more intensive training methods can be resource-intensive and limit EBP spread. These limitations in resources have led to development of efficient, scalable, and lower cost training methods such as online training and blended learning models, which incorporate elements of both online training and expert consultation. Such an approach may increase provider access [17], especially where expert training is limited, such as in rural areas and developing countries.

To date, the ability to reach conclusions regarding the most effective EBP training methods has been hampered by methodological limitations (e.g., pre-post, non-controlled studies) and the use of self-assessment of provider skill acquisition and competence, which is not always consistent with objective measurement of provider behavior [15]. Moreover, studies on EBP training have not routinely reported client outcomes, and most research has focused exclusively on mental health providers, although it is becoming increasingly common for non-mental health professionals (e.g., primary care providers, nurses) to deliver brief EBPs for mental health conditions such as alcohol misuse [18]. Evaluating the evidence for EBP training methods is an important step in determining the effectiveness and comparative effectiveness on implementation, system, and client outcomes, with the goal of increasing provider skill, improving client outcomes, and containing training costs. Finally, while the implicit goal of EBP training has been to increase the reach of these treatments, the psychotherapy training literature has largely developed in parallel to implementation science. Historically, EBP training and clinical supervision has focused on building providers’ expertise in the EBP and has lacked implementation strategies to integrate and sustain the clinical innovation. Mapping strategies used in EBP training and consultation/supervision and defined implementation strategies [14] will facilitate comparisons across clinical innovations and identify future EBP training research directions.

### Objectives

We conducted a systematic review of randomized controlled trials that evaluated EBP training methods to determine the effectiveness and comparative effectiveness of EBP training modalities (e.g., in-person training with consultation, blended-learning, online only) on implementation outcomes (e.g., provider EBP knowledge, adherence, competence, and fidelity as well as costs such as financial resources and provider time). We also examined the effect of EBP training on client outcomes (e.g., symptom scores).

## Methods

Details of the methods for our systematic review are described below and correspond with the Preferred Reporting Items for Systematic Reviews and Meta-Analyses (PRISMA) guidelines (see supplemental material for PRISMA checklist) [19]. The protocol for this review is registered in the international prospective register of systematic reviews (PROSPERO; CRD42018093381).

### Searches

We searched MEDLINE (Ovid) and PsycINFO for studies published from 1990 through June 2019. See Table 1 for the full electronic search strategy used for MEDLINE. We hand-searched the reference lists of relevant systematic reviews to identify additional studies that may have been missed in our electronic search.

**Table 1:** MEDLINE (Ovid) search strategy

Block 1: Training search terms	
1. psychotherapy training.mp.	
2. professional consultation.mp.	
3. Psychotherapy/ed, is, mt, og, st, tu, ut	
4. (evidence based train*).mp.	
5. (therapist* train*).mp.	
6. (blended learn*).mp.	
7. (online train*).mp.	
8. exp clinical competence/ or (clinical competence).mp	
9. or/1-8	
Block 2: Therapy search terms	
10. exp cognitive therapy/ or cognitive behavior* therapy.mp.	
11. ((behavior* or exposure or implosive) adj therapy).mp.	
12. motivational interviewing.mp.	
13. psychodynamic psychotherapy.mp.	
14. or/10-13	
Block 3: Condition search terms	
15. ((anxiety or depress*) adj disorder*).mp.	
16. ((substance or substance-related) adj2 disorder*).mp.	
17. ((eat*) adj disorder*).mp.	
18. bipolar disorder*.mp.	
19. schizophrenia.mp.	
20. (child behavior* disorder*).mp.	
21. (developmental disorder*).mp.	
22. externalizing.mp.	
23. internalizing.mp.	
24. (emotion* disorder*).mp.	
25. (behavior* disorder*).mp.	
26. or/15-25	
Block 4: Combining terms adding limits	
27. 9 and (14 or 26)	
28. limit 27 to (yr="1990 -Current" and english)	
29. exp Meta-Analysis/	
30. (metaanaly* or meta-analy* or meta analy*).mp.	
31. (systemat* adj (review or overview)).mp.	
32. 29 or 30 or 31	
33. (random* adj3 trial).mp or exp Randomized Controlled Trial/	
34. (random* adj (allocat* or assign* or enroll*)).mp.	
35. randomi#ed.mp.	
36. 33 or 34 or 35	
37. 28 and 32	
38. 28 and 36.	

### Study inclusion and exclusion criteria

Studies were potentially eligible for inclusion if they evaluated methods used to train providers to deliver EBPs to individuals with mental health diagnoses. We included randomized controlled studies if (1) they were published in a peer reviewed journal, in English, between 1990 and June 2019 (date range was in line with Herschell and colleagues’ (2010) review as prior reviews summarized previous research [20], and EBPs were still largely in development at this time); (2) the intervention that providers were trained in was an EBP (determined through consensus by the first and last authors), using available guides [21, 22]; (3) EBPs were designed to treat individuals who had a mental health condition; (4) authors included information on the EBP training (e.g., method used and components of training); and (5) relevant training outcomes were reported (see provider-level outcomes below). We excluded studies that did not evaluate provider training in the EBP. Study inclusion criteria was deliberately kept broad with respect to provider characteristics/training, EBPs which were the focus of trainings, and client mental health conditions in order to maximize the number of relevant studies given the relatively small body of controlled research on training methods to date.

Studies must have reported at least one of the following provider-centered outcomes to be eligible for the review: (a) provider satisfaction with training, (b) provider treatment knowledge, (c) provider skill acquisition, (d) provider adherence; (e) provider competence, (f) provider fidelity, or (g) EBP adoption (e.g., use of EBP after the training was completed). Additionally, the following outcomes were examined if reported: (a) training cost and (b) client outcomes post-EBP training. For included studies, the provider population (e.g., individuals receiving the EBP training) could include any health professional (e.g., psychologist, social worker, primary care physician, addiction specialist, etc.) delivering an EBP. If clients were included in the study, the client population (e.g., individuals receiving the EBP) was defined as individuals receiving the mental health treatment by study providers. All EBP training modalities (e.g., workshops, online training) were eligible, including studies that manipulated post-initial training consultation. EBP training modalities could be compared to other active training conditions, training controls, or waitlist conditions, and they needed to assess provider outcomes at least pre- and post-training. Additional follow-up was not required for inclusion.

### Study quality assessment

We assessed risk of bias for individual studies included in the review using a modification of the Cochrane risk of bias criteria [23]. The risk of bias rating was based on the following categories: random sequence generation, allocation concealment, masking of participants and/or personnel, attrition bias, and selective outcome reporting. Categories which were not described adequately in the paper were given an “unclear” designation (considered a medium risk of bias). Given the nature of the studies included in the review (e.g., studies in which providers are trained in EBPs), we considered it difficult for studies to mask participants to training condition. Thus, studies could still qualify as having a low risk of bias rating for masking if personnel involved in outcome assessment (e.g., simulated clients; adherence coders) were masked to the participants’ condition. Regarding attrition bias, we categorized studies as having low risk of bias if they had less than 10% incomplete provider outcome data due to attrition, medium if they had between 10 and 20%, and high if they had more than 20%. For overall risk of bias, studies were categorized as low if they were rated as low for random sequence generation and masking, low or medium for attrition, and had no categories rated as high. Studies were rated as having overall medium risk of bias if they had no more than one category rated as high, and studies were rated as having an overall high risk of bias if they had more than one category rated as high.

Finally, the overall strength of evidence for each outcome was evaluated using the five required domains outlined in the Agency for Healthcare Research and Quality rating system [24]. Specifically, for each outcome, the quality of evidence rating was based on studies’ limitations (risk of bias), consistency in direction/magnitude of effects, directness (interventions relation to clinical health outcome), precision (degree of certainty regarding effect estimate), and potential reporting bias. The overall strength of the evidence was then rated as high, moderate, low, or insufficient based on aggregation of these five domain ratings. Risk of bias and quality of evidence were rated by two independent reviewers and inconsistencies in ratings were discussed to reach consensus.

### Data extraction strategy

Two trained reviewers independently reviewed all study abstracts to identify potentially eligible articles based on the *a priori* criteria listed above. Studies underwent full-text review if either reviewer thought it was relevant to the review’s aims and appeared to meet inclusion criteria. The studies selected for full-text review were again evaluated independently by two reviewers. Studies were included in the systematic review if both investigators deemed them to meet the inclusion criteria. Investigators discussed any discrepancies regarding their inclusion/exclusion decision and consulted with a third member of the review team if necessary.

#### Extraction of data items

Data extraction was completed by one reviewer and verified by a second. We extracted the following elements from each study:

1. Study characteristics including training methods evaluated, EBP type and mental health condition, provider and client characteristics, assessment measures, and assessment timeframe
2. Provider outcomes including satisfaction with training, treatment knowledge, skill acquisition, adherence, competence, fidelity, and adoption
3. Cost of training
4. Client clinical outcomes (e.g., symptom scores)

We also extracted the components (e.g., role-play, expert consultation, video/audio review) of each training method evaluated in the studies included in the review.

### Data synthesis and presentation

We generated descriptive statistics (i.e., count and percent) of articles reporting on each EBP training modality as well as each provider-centered outcome. Given the heterogeneity of the training methods, assessment measures, and providers and clients, we described the results using a narrative/descriptive synthesis instead of a quantitative analysis.

## Results

**Fig. 2:**
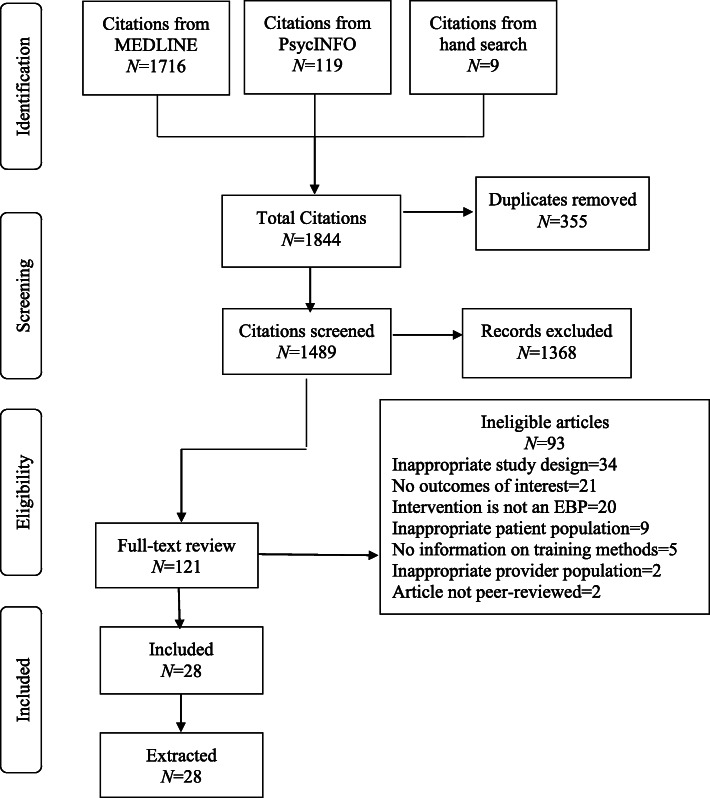
Literature flow

The literature flow is reported in Fig. 2. Our electronic and hand searches yielded a total of 1844 studies. After removal of duplicates (*n* = 355), 1489 studies underwent abstract review. Of those studies, 1368 studies were subsequently excluded, leaving 121 articles for full-text review, including nine studies identified through a hand search of recent systematic reviews and reference lists of included studies. Of those, 93 were excluded for a total of 28 eligible and included studies [25–52].

Table 2 includes a description of the characteristics of included studies and Table 3 characterizes discrete implementation strategies used in the EBP training methods included in each study. Additional study information and risk of bias for each study are reported on Appendix Table 1. Overall, there was considerable heterogeneity between studies. Three quarters (75%) of studies were conducted in the USA, with the remainder in multi-country or international settings. Samples sizes of providers ranged from *n* = 30 to *n* = 181, with 39% of studies having over 100 providers and 32% having less than 50 providers. Provider characteristics (e.g., degree, previous experience with EBP; Appendix Table 1) also varied widely between studies and 50% of the studies included providers who were unlicensed/trainees. Slightly over half (57.1%) of studies included an objective assessment of provider behavior (e.g., rating of provider’s adherence in a simulated client interaction); the remainder of the studies (with the exception of one which reported on client outcomes only) used an EBP knowledge test and/or client report/provider self-report of provider behavior to assess the effects of training. The definition and measurement of provider outcomes varied between studies (Appendix Table 1) and follow-up timeframe for assessment of provider outcomes ranged between studies from pre-training to immediately post-training only to pre-training to 12-months post-training. Regarding type of EBP, 36% of studies evaluated training for a variant of Cognitive Behavioral Therapy (e.g., a range of Cognitive Behavioral Therapy protocols designed to treat different mental health conditions), with Motivational Interviewing the second most common (21% of studies). The two most common mental health conditions the EBP was designed to treat was mood disorders (depression and/or anxiety; 39.2%), followed by substance use disorders (21.4%). Six eligible studies were rated as low risk of bias, 21 medium risk of bias, and one high risk of bias. Regarding implementation strategies included in EBP training methods, the most common strategies were making the training dynamic and providing clinical supervision, with only a few training methods including other strategies (e.g., relay of clinical data to providers, learning collaborative, etc.)

**Table 2:** Descriptive characteristics of included studies (*k =* 28)

Characteristic	*n* and proportion (%) of included studies
Provider sample size	
*N* < 50	9 (32.1%)
*N* = 50–100	8 (28.6%)
*N* > 100	11 (39.2%)
Provider sample included trainees/unlicensed individuals	14 (50.0%)
Study included objective assessment of provider behavior	16 (57.1%)
Study included knowledge test/self-report only	11 (39.2%)
Study included patient outcomes only	1 (3.6%)
EBPs	
Cognitive behavioral therapy variant	10 (35.7%)
Motivational interviewing	6 (21.4%)
Exposure therapy	4 (14.3%)
Dialectical behavior therapy	3 (10.7%)
Behavioral activation	2 (7.1%)
Contingency management	1 (3.6%)
Cognitive processing therapy	1 (3.6%)
Trauma-focused cognitive behavioral therapy	1 (3.6%)
Mental health condition EBP designed for	
Depression and/or anxiety disorders	11 (39.2%)
Substance use disorders	10 (35.7%)
Borderline personality disorder	3 (10.7%)
Posttraumatic stress disorder	3 (10.7%)
Eating disorders	1 (3.6%)

*EBP* evidence-based psychotherapy

Cognitive behavioral therapy variant refers to a range cognitive behavioral therapy protocols designed to treat different mental health conditions

**Table 3:** Discrete ERIC implementation strategies (Powell et al. 2015) used in EBP training methods in included studies

Study (first author and year)	EBP Mental health condition	Training methods evaluated	Implementation strategies					
			Distribute educational materials	Facilitate relay of clinical data to providers	Make training dynamic	Provide clinical supervision	Learning collaborative	Other
Bearman 2017 [25]	Cognitive behavioral therapy Youth depression	In-person workshop + experiential supervision			X	X		X (supervision dynamic)
		In-person workshop + supervision as usual			X	X		
Beidas 2012 [26]	Cognitive behavioral therapy, Coping Cat protocol Youth anxiety	In person didactic workshop + virtual consultation				X		
		Online training + virtual consultation				X		
		In-person didactic/experiential workshop + virtual consultation			X	X		
Bennett-Levy 2012 [27]	Cognitive behavioral therapy Depression, panic disorder, with agoraphobia, generalized anxiety disorder	Online training			X			
		Online training + supportive calls			X			X (supportive calls to providers)
Chu 2017 [28]	Cognitive behavioral therapy Youth anxiety	Online training + online expert streaming consultation				X (watch streamed)		
		Online training + in-person peer consultation					X	
		Online training + fact sheet self-study	X					
Cohen 2016 [29]	Trauma-focused cognitive behavioral therapy PTSD and depression	Online training + online consultation course			X	X (automated)		
		Online training + in person workshop + phone consultation			X	X		
Cooper 2017 [30]	Cognitive behavioral therapy Eating disorders	Online training			X			
		Online training + supportive calls			X			X (supportive calls to providers)
Dimeff 2009 [31]	Dialectical behavior therapy Borderline personality disorder	Online training			X			
		In-person workshop						
		Treatment manual	X					
Dimeff 2011 [32]	Dialectical behavior therapy Borderline personality disorder	Online training			X			
		Placebo online training						
		Treatment manual	X					
Dimeff 2015 [33]	Dialectical behavior therapy Borderline personality disorder	Online training			X			
		In-person workshop			X			
		Treatment manual	X					
Fu 2015 [34]	Motivational interviewing Smoking cessation	In-person workshop			X			
		In-person workshop + booster sessions + peer coaching			X	X	X	
Gega 2007 [35]	Exposure therapy Phobias	Online training						
		In-person workshop						
Harned 2011 [36]	Exposure therapy Anxiety disorders	Online training			X			
		Online training + motivational interviewing			X			X (motivational component for providers to adopt EBP)
		Placebo online training						
Harned 2014 [37]	Exposure therapy Anxiety disorders	Online training			X			
		Online training + motivational enhancement			X			X (motivational component for providers to adopt EBP)
		Online training + motivational enhancement + learning community					X (with expert)	
Henggeler 2008 [38]	Contingency management Marijuana abuse	In-person workshop			X			
		In-person workshop + Intensive Quality Assurance			X	X		
Hubley 2015 [39]	Behavioral activation Depression	Online training						
		Online placebo control training						
Larson 2013 [40]	Cognitive behavioral therapy Substance abuse	Online training + supervision			X	X		
		Training manual + supervision	X			X		
Martino 2011 [41]	Motivational interviewing Substance abuse	In person workshop by expert + consultation		X		X		
		In person workshop by trainee + consultation		X		X		X (train-the-trainer)
		Treatment manual + didactic materials	X					
McDonough 2002 [42]	Exposure therapy Phobias	In-person lecture + online training			X			
		In-person lecture + in-person group tutorial			X			
Miller 2004 [43]	Motivational interviewing Substance abuse	In-person workshop			X			
		In-person workshop + audio review and feedback		X	X			
		In-person workshop + coaching			X	X		
		In-person workshop + audio review and feedback + coaching		X	X	X		
		Training manual + didactic videotapes	X		X			
Monson 2018 [44]	Cognitive processing therapy Posttraumatic stress disorder	In-person workshop						
		In-person workshop + consultation				X		
		In-person workshop + consultation with session audio review		X		X		X (relay of clinical data in supervision)
Moyers 2008 [45]	Motivational interviewing Substance abuse	In-person workshop						
		In-person workshop + feedback + consultation		X		X		
		Training manual + didactic videotapes	X					
Puspitasari 2017 [46]	Behavioral activation Depression	Distance workshop			X			
		Online training						
Rakovshik 2016 [47]	Cognitive behavioral therapy Panic disorder w/ agoraphobia, obsessive-compulsive disorder	Online training + consultation worksheet	X		X			
		Online training + consultation worksheet + remote supervision			X	X		
		Delayed training control						
Rawson 2013 [48]	Cognitive behavioral therapy Stimulant dependence	In-person workshop + in-person coaching			X	X		
		Distance workshop + telephone coaching			X	X		
		Training manual + orientation	X					
Ruzek 2014 [49]	Cognitive behavioral therapy skills Posttraumatic stress disorder	Online training			X			
		Online training + telephone consultation			X	X		
		No training control						
Smith 2012 [50]	Motivational Interviewing Substance abuse	In-person workshop			X			
		In-person workshop + session audio review		X	X			
		In-person workshop + live teleconferencing supervision			X	X		
Weingardt 2006 [51]	Cognitive behavioral therapy Cocaine addiction	In-person workshop						
		Online training						
		Delayed training control						
Zatzick 2014 [52]	Motivational Interviewing Problem drinking	In-person workshop + feedback + coaching		X		X		
		No training						

Appendix Table 2 includes an overview of the main findings reported in each study including effect and *p* values sizes as available. Appendix Tables 3a-3c include data for each outcome extracted from each study. Differences (or lack thereof) between conditions summarized below are based on statistical significance. The synthesis of the included studies’ results, described below, is organized by implementation outcomes [12] examined in the review. We categorized training conditions as (1) no training/self-study (participants in these conditions were randomized to waitlist, given a EBP treatment manual and/or didactic materials to study independently, or engaged in a placebo training, in which content unrelated to the EBP interventions was presented); (2) active training (participants were randomized to any type of active training such as in person workshop, online training, distance learning, etc.). Active trainings could be either instructor-led, self-paced, or a combination of the two; or (3) active training plus consultation (participants were randomized to receive additional consultation after their initial training). A few studies in category 3 compared different consultation methods after participants received the same initial training. We describe the results for each outcome by comparisons of the following training conditions: active training compared to no training/self-study, active training comparison, and active training compared to active training plus consultation.

### Implementation outcomes

#### Provider satisfaction with training

Ten studies (36%) evaluated provider satisfaction with training [26, 28, 31–33, 35–37, 42, 46].

##### Active training compared to no training/self-study/placebo

Based on four studies [31–33, 36], we found moderate strength of evidence that more active training conditions resulted in greater satisfaction compared to self-guided study of treatment manual/training materials or placebo online training control condition. Specifically, one study found that both an in-person and online training had higher satisfaction ratings than self-study of the treatment manual [31]. Another found that the in-person training, but not the online training, was rated more satisfactory than self-study of the treatment manual [33]. The two other studies looked at two indices of satisfaction: acceptability (e.g., helpfulness of the training materials) and usability (e.g., ease of navigation through the training material). Both studies found that active online trainings were rated more acceptable than a placebo online training [32, 36]. Regarding usability, one study found no difference between the active online training conditions and the placebo online training condition [36]. The other study found that both the active and placebo online trainings were rated as more usable than the training manual [32].

##### Active training comparison

Seven studies [26, 31, 33, 35, 36, 42, 46] compared provider satisfaction with different active training methods. Overall strength of evidence was low for any active training condition resulting in greater satisfaction than another. Four studies [31, 35, 36, 46], two of which compared online and in-person training, one which compared online training to an enhanced online version, and one which compared distance workshop (remote training with live instructor) to online training, found no differences in satisfaction between active training methods. Three studies [26, 33, 42] found that participants were more satisfied with in-person training compared to online training.

##### Active training compared to active training plus consultation

Two studies [28, 37] examined provider satisfaction regarding consultation after training. Results were mixed and strength of evidence rated as insufficient. One study found no difference in satisfaction between two active consultation conditions and a no-consultation control [28]. Another study found that the motivational enhancement element of the training intervention, aimed at addressing providers’ potential attitudinal barriers to learning and using the EBP, was rated as more acceptable when it was combined with an online learning collaborative [37].

#### Provider EBP treatment knowledge

Sixteen studies (57%) evaluated provider EBP treatment knowledge [26–28, 30–37, 39, 42, 48, 49, 51].

##### Active training compared to no training/self-study/placebo

Eight studies compared change in treatment knowledge between one or more active training conditions and a no-training, self-guided study of treatment manual/training materials, or placebo online training control condition [31–33, 36, 39, 48, 49, 51]. All studies found at least one active training condition resulted in greater gains in treatment knowledge than the control condition (moderate strength of evidence).

##### Active training comparison

Eleven studies compared gains in treatment knowledge between different active training methods [26, 27, 30, 31, 33, 35–37, 42, 48, 51]. Strength of evidence was rated low for any active training method resulting in increased treatment knowledge over another active method. Nine found no differences in treatment knowledge between active training conditions [26, 35–37, 42, 48, 51], including five studies that compared online and in-person training [26, 35, 42, 48, 51], two studies that compared online training to an enhanced online version [36, 37], and two studies that compared online training to online training with supportive phone calls [27, 30]. Dimeff (2009, 2015) did find a difference between active training conditions: specifically, online training conditions resulted in greater increases in treatment knowledge than expert-led, in-person training [31, 33].

##### Active training compared to active training plus consultation

Four studies compared the additive effects of consultation on treatment knowledge beyond initial training or compared different consultation methods and overall strength of evidence was low [28, 34, 37, 49]. One study found that treatment knowledge was similar between participants receiving different types of consultation (online expert-led consultation, in-person peer consultation, or self-study of fact sheet control); notably, participants in all three conditions had *decreases* in treatment knowledge pre- to post-consultation/self-study of fact sheet [28]. Three studies found that adding expert consultation (two after online training and one after in-person training) led to greater increases in treatment knowledge in at least some domains compared to completing the initial training only [34, 37, 49].

#### Provider skill acquisition/adherence

Fifteen studies (54%) evaluated provider skill acquisition/EBP adherence [26–29, 31, 34, 38, 40, 41, 43, 45, 48–50, 52].

##### Active training compared to no training/self-study/placebo

Six studies compared one or more active training conditions to a no-training or self-guided study of treatment manual/training materials control condition [41, 43, 45, 48, 49, 52]. We found a moderate strength of evidence that more active training resulted in improved adherence. Five out of six studies found at least one active training condition resulted in greater gains in EBP adherence than the control condition [41, 43, 48, 49, 52]; one study found no difference in skill acquisition between participants who attended an in-person workshop and those who engaged in self-study of training materials [45].

##### Active training comparison

Five studies compared gains in skill acquisition/adherence between different active training methods and all found no difference in adherence between active training conditions [26, 27, 40, 41, 48] (low strength of evidence).

##### Active training compared to active training plus consultation

Seven studies compared the additive effects of consultation beyond initial training or compared different consultation methods on skill acquisition/adherence [29, 34, 38, 43, 45, 49, 50]. We found low strength of evidence that active training with consultation resulted in greater EBP adherence than active training alone. Two studies found that adding consultation (up to six, 30-min consultation calls) did not lead to increases in EBP adherence compared to an in-person workshop alone [43, 45]. Five studies found that at least one form of consultation did lead to greater gains than training alone (consultation dosage in these studies ranged from five sessions over 5 weeks to weekly sessions over 6 months) [29, 34, 38, 49, 50]. Of note, one of these studies used provider self-report [29] and one used client report of provider behavior [38] to measure adherence, which may not be as valid as objective coding of provider behavior. Additionally, Ruzek (2014) found that online training plus consultation (up to six, 45- to 60-min consultation calls) led to increased adherence in only one out of three of the EBP skills taught compared to online training only [49].

#### Provider competence

Fourteen studies (50%) evaluated change in provider competence [25, 26, 31, 33, 34, 37, 41, 43, 45–48, 50, 52].

##### Active training compared to no training/self-study/placebo

Eight studies [31, 33, 41, 43, 45, 47, 48, 52] compared one or more active training conditions to a no-training or self-guided study of treatment manual/training materials control condition. Overall strength of evidence was rated low. Of those eight, five found that at least one active training condition led to greater increases in provider competence than no training/self-guided study [41, 43, 45, 48, 52]. Specifically, all five studies found that an expert-led in-person workshop was superior to self-guided study in increasing provider competence. Three studies, however, found no difference between at least one active training condition (online training or expert-led in-person training) and no-training/self-guided study control condition [31, 33, 47].

##### Active training comparison

Seven studies compared the effects of different active training methods on provider competence and strength of evidence for the superiority of one active training method improving competence over another was low [26, 31, 33, 37, 41, 46, 48]. Five of the seven studies found no differences in change in provider competence between training methods (three studies compared in-person to online training, one study compared online training to an enhanced online training, and one study compared in-person to distance learning). Participants in all conditions had an increase in competence pre- to post-training/follow-up [26, 31, 33, 37, 48]. Two studies found differences between active training conditions: Puspitasari (2017) found that an expert-led, live distance (online) training led to greater gains in Behavioral Activation skills competence compared to a self-paced online training [46]. Martino (2011) found that an expert-led in-person training and consultation, compared to “train-the-trainer”-led in-person training and consultation, led to greater increases in fundamental competence of Motivational Interviewing in role-played sessions, but not client sessions, from pre-training to 12-week follow-up [41].

##### Active training compared to active training plus consultation

Eight studies compared the additive effects of consultation beyond initial training or compared different consultation methods [25, 34, 37, 43, 45, 47, 48, 50]. Overall strength of evidence was rated as low. Five studies compared a training plus consultation condition to a no consultation (initial training only) condition [34, 37, 43, 45, 47]. Three of these studies found that adding consultation (ranging from three to eight consultation sessions) in addition to training (one study post in-person training, two post online training) resulted in greater increases in provider competence compared to training only [34, 37, 47]. However, two studies found that consultation did not result in additional benefit regarding provider competence beyond the initial in-person training [43, 45], although in Miller (2004), only the conditions with consultation had average competency scores at the clinical proficiency standard.

Three studies compared the effect of different active consultation conditions on provider competence. One study found no differences between consultation conditions (practice feedback, individual consultation sessions, or both) [43]. Two studies found differences: Bearman (2017) found an experiential, active consultation led to greater increases in provider competence and EBP expertise compared to traditional consultation [25], and Rawson (2013) found that in-person consultation led to greater increases in provider competence than telephone consultation and a higher level of competence at a 24-week follow-up [48].

#### Provider fidelity

Only one study evaluated change in providers’ EBP fidelity (a composite adherence and competence rating) so strength of evidence was rated as insufficient. Bearman 2017 found enhanced supervision after an in-person workshop, which utilized experiential learning (e.g., modeling and role-plays) and provided feedback on session recordings, resulted in greater increases in provider EBP fidelity compared to supervision as usual post in-person workshop [25].

#### EBP adoption

Nine studies (32%) evaluated adoption of the EBP in clinical practice. All of the studies measured adoption via provider self-reported use of EBP techniques through teaching and/or with clients [27, 28, 31–33, 36, 37, 46, 49].

##### Active training compared to no training/self-study/placebo

Based on five studies comparing adoption of EBP into clinical practice between one or more active training conditions and a no-training, self-guided study of treatment manual/training materials, or placebo online training control condition [31–33, 36, 49], active training may not have increased EBP adoption (low strength of evidence). Four of the five studies found that the active training condition did not result in greater adoption of EBP components than control (assessment of adoption ranged from immediately post-training to 90-days post-training) [31, 33, 36, 49]. Dimeff (2011) found that online training led to greater self-reported teaching and/or use of EBP skills learned compared to online placebo training at all four follow-up timepoints (2-weeks to 15-weeks post-training); however, it only led to greater teaching/use of EBP skills at one timepoint compared to self-study of the treatment manual [32].

##### Active training comparison

Five studies compared EBP adoption between different active training methods [27, 31, 33, 37, 46]. None of the five studies found a difference in adoption between the active training conditions (three compared online to in-person training, two compared online training to an enhanced or supported online training) (low strength of evidence).

##### Active training compared to active training plus consultation

Three studies compared the additive effects of consultation beyond initial training or different consultation methods on EBP adoption [28, 37, 49]. None found a difference between conditions in regard to EBP adoption (low strength of evidence).

#### Costs

Three studies (11%) reported on EBP training costs. Rawson (2013) presented costs for provider training in an EBP (Cognitive Behavioral Therapy for stimulant dependence) for the three training conditions examined. The highest cost training method was in-person workshop plus in-person consultation ($1485 per participant), followed by distance workshop plus telephone consultation ($768 per participant), followed by providing participants with the training manual and an orientation session ($145 per participant) [48]. Two other studies presented the costs associated with the active training condition studied (online training in Cognitive Behavioral Therapy: $440 per participant [27]; in-person training plus feedback and consultation in Motivational Interviewing: $4300 per site [52]). No studies examined the cost of training as it related to the comparative effectiveness of the training methods studied.

### Client outcomes

Three studies (11%) reported on client clinical outcomes in relation to EBP training [29, 44, 52] and strength of evidence was rated as insufficient. One study found that clients in sites where providers were trained in the EBP (Motivational Interviewing) had a greater reduction in hazardous drinking scores in the year after hospitalization than clients in sites where providers were not trained [52]. Another study found that, after controlling for provider effects, clients were more likely to complete treatment (Trauma-focused Cognitive Behavioral Therapy) from providers who were randomized to complete online training and receive consultation compared to providers who were randomized to complete online training only [29]. The third study found that an expert-led in-person workshop in an EBP (Cognitive Processing Therapy) plus standard consultation resulted in greater reductions in client PTSD symptoms compared to an in-person workshop only. However, no difference was found in client outcomes between in-person workshop only and in-person workshop plus consultation with audio review [44].

## Discussion

Active EBP training probably leads to greater increases in provider EBP knowledge and adherence and providers are probably more satisfied with training compared to no training, placebo training, or self-study of treatment materials. However, it is unclear if these provider-level implementation outcomes (e.g., satisfaction, treatment knowledge) translate to more effective delivery of EBPs and better client outcomes.

Findings were mixed regarding whether EBP training led to greater increases in competence, and no difference was found in EBP adoption between providers who received EBP training and those who did not. Thus, it may be that although training in EBPs leads to increases in providers’ ability to employ foundational elements of EBP delivery (e.g., treatment knowledge and skill acquisition/adherence), it does not result in higher quality delivery of the treatment (e.g., competence). Competent, skillful delivery of the treatment may be more difficult to teach in a relatively brief EBP training context, and training in these more nuanced therapeutic skills may need to begin earlier in providers’ education or be further developed through additional practice, consultation, or other supports. Additionally, no specific EBP training modality (e.g., in-person training, online training) was more effective than another with regard to increasing provider EBP knowledge, skill acquisition/adherence, competence, adoption, or satisfaction.

In contrast to the general conclusions of previous reviews [15, 16], the additional benefit of consultation beyond initial EBP training was inconclusive. Specifically, no difference was found in EBP adoption between those who received active training and those who received active training plus consultation, and findings were mixed regarding the additive benefit of consultation on provider EBP knowledge, adherence/skill acquisition, competence, and satisfaction. Our findings may differ from the previous reviews [15, 16] in that, in addition to including newer research, only studies using randomized controlled designs were included which, taken together, provide inconclusive support for the additional contribution of consultation. Differences in consultation “dose” and intensity may have contributed to these mixed findings. Individual studies point to elements of consultation that may be particularly effective, such as performance feedback, modeling, and role-playing [25], and others that may detract, such as audio review in a group consultation setting [44]. However, more research is needed to identify effective consultation practices that lead to providers’ continued skill development over time. Moreover, the finding that providers trained in EBPs and provided with additional consultation may not be more likely to adopt EBPs than those who are not trained or only engage in self-study is concerning. Underutilization of EBPs is problematic considering the extensive resources employed to train providers in EBPs in an effort to increase access to effective treatment. Prior work with other types of clinical innovations have consistently demonstrated that educational workshops alone is insufficient to impact complex behavior change [53] and implementation strategies beyond provider training are needed for successful implementation. Making the training dynamic and post-training clinical supervision/consultation were the most widely used strategies and most studies reported using few other implementation strategies [14]. These findings add to that literature by clarifying that specific implementation strategies beyond those focused on enhancing clinical knowledge and skill may be needed. Current initiatives, such as the VHA’s PTSD Consultation Program, which provides ongoing, free continuing education and consultation to ensure high-quality EBP delivery for PTSD, is a recent example of an EBP training initiative broadening clinical training to encompass more implementation strategies; evaluation of implementation outcomes of such programs is warranted [54]. To ensure continued utilization of EBPs after training and consultation have ended, more focus is needed on modifying providers’ negative beliefs about EBPs and utilizing novel behavioral strategies to increase provider adoption of EBPs [55, 56]. Additionally, further study is necessary to understand the larger contextual factors (e.g., health care systems, organizational, and team factors) that may influence the use of EBPs [57].

While this review captured a broad range of studies evaluating different EBP training methods, there was substantial heterogeneity among studies, making it difficult to compare results across studies or draw conclusions about the effectiveness of specific training methods. Studies included in the review evaluated training methods for different EBPs, included providers at different training levels and with different levels of exposure to the EBP, and evaluated a diverse set of training and consultation methods. Future research should examine the effect of provider background and training, EBP type, and client mental health conditions, on EBP training outcomes.

Furthermore, the definitions and measurement of the provider-centered outcomes differed across studies (see Appendix Table 1 for a detailed description of these elements for each study). While the studies included in this review all utilized a strong research design (i.e., randomized controlled design), and with one exception [47], were rated as having a low to medium risk of bias, the strength of evidence for outcomes of interest was often limited due a lack of consistency in the direction of effects (e.g., some studies finding statistically significant differences between training conditions and others having null results). Additionally, strength of evidence was rated as insufficient for some outcomes due to the low number of studies (e.g., one or two) evaluating the outcome, or because studies defined outcomes differently, and thus, had limited convergent evidence. Thus, a lack of consistency in direction of effects across studies may be accounted for, in part, by variability in construct definitions and measurement rather than by effectiveness of training method. Future research in this area should focus on the development and use of uniform measures of EBP treatment adherence and competence whenever possible. Finally, the follow-up assessment timeframes of many of the studies included in the review were relatively short, potentially skewing findings about provider gains. Longer-term follow-up would allow for assessment of durability of provider gains over time. Importantly, recent research has demonstrated that providers’ EBP adherence is not static and often varies between sessions [58]; thus, assessment of provider adherence and competence may be best evaluated using multiple data points over the course of treatment. Given the limitations of the current body of evidence, conclusions about the most effective EBP training methods should be drawn with caution.

Notably, the majority of studies included in the review did not measure several important outcomes. First, there is an overall dearth of research evaluating the effect of training on client clinical outcomes, the outcome of greatest importance. Secondly, no studies evaluated changes in implementation or client outcomes from different training methods with regards to costs, such as instructor and provider time spent in initial training activities and post-training consultation. Extrapolating from costs reported across studies, in-person workshops followed by in-person consultation may be over three times more expensive than online training and over ten times more expensive than providing treatment manuals [27, 48]. Future studies should report costs for each training method to allow for analysis of the impact of increasingly more resource-intensive training methods (e.g., expert-led in-person training with ongoing consultation) on provider and client outcomes achieved with higher levels of resource investment. This would allow stakeholders, especially those in low-resourced organizations (e.g., community mental health clinic directors), to determine which training initiatives are worth the investment.

It is important to note the limitations of the review itself. Limitations of the review include the use of two databases and a non-exhaustive list of training terms or psychotherapy approaches (e.g., did not include family therapies or parenting interventions), which may have resulted in the omission of relevant studies.

## Conclusions

In summary, there is evidence that EBP training leads to short-term gains in the provider outcomes of EBP knowledge acquisition, adherence, and satisfaction compared to no training, placebo training, or self-study of treatment materials. Results were mixed for the effect of EBP training on provider competence and no effect was found for EBP adoption. No EBP training method demonstrated clear superiority over others. Additionally, the additive benefit and essential elements of post-training consultation are unclear. Given the absence of strong evidence regarding the most effective training methods, health systems should consider other factors when selecting an EBP training method, such as organizational costs (e.g., financial resources, provider productivity). Additionally, both providers and organization stakeholders should be aware that participation in an EBP training may not be sufficient to ensure delivery of high-quality EBPs to clients.

## Supplementary information

**Additional file 1: **Appendix: **Table 1.** Study descriptions and Cochrane risk of bias rating. **Table 2** Overview of included studies’ findings (*k* = 28). **Table 3a** Study outcomes: participant adherence/EBP skill acquisition, competence, and fidelity. **Table 3b** Study outcomes: participant satisfaction, EBP treatment knowledge, and EBP adoption. **Table 3c** Study outcomes: client clinical outcomes and costs of training.

## Data Availability

All articles included in this systematic review are publicly available.

## Abbreviations

EBP: Evidence-based psychotherapy
NCTSN: National Child Traumatic Stress Network
PRISMA: Preferred Reporting Items for Systematic Reviews and Meta-Analyses
PROSPERO: International Prospective Register of Systematic Reviews
SAMHSA: Substance Abuse and Mental Health Services Administration
VA: Department of Veterans Affairs
VHA: Veterans Health Administration
