# Supplementary material for: Effectiveness of training methods for delivery of evidence-based psychotherapies: a systematic review
Source: Implement Sci. 2020 May 27;15:40. doi: 10.1186/s13012-020-00998-w (PMC7251851; doi:10.1186/s13012-020-00998-w)
Supplement: Supplementary file 1 — Additional file 1: Appendix: Table 1. Study descriptions and Cochrane risk of bias rating. Table 2 Overview of included studies’ findings (k = 28). Table 3a Study outcomes: participant adherence/EBP skill acquisition, competence, and fidelity. Table 3b Study outcomes: participant satisfaction, EBP treatment knowledge, and EBP adoption. Table 3c Study outcomes: client clinical outcomes and costs of training. [file 13012_2020_998_MOESM1_ESM.docx]

**Appendix**

***Note: All abbreviations used in tables are listed at the end of the appendix.***

**Table 1. Study Descriptions and Cochrane Risk of Bias Rating**

| **Study**  **Location**  **Setting**  **EBP Evaluated**  **MH Condition** | **Description of Training Methods** | **Participant Characteristics (*N*)**  **Inclusion/Exclusion criteria**  **Previous Experience with EBP**  **Discipline**  **Demographics** | **Client Characteristics** | **Assessment Measures**  **Assessment Timeframe** | **Cochrane Risk of Bias** |
| --- | --- | --- | --- | --- | --- |
| Bearman 2017  *Location*:  United States  *Setting:*  Mental health trainees, university setting  *EBP evaluated*:  Cognitive Behavioral Therapy (CBT) (cognitive restructuring technique)  *MH condition*:  Youth depression | *Training format:*  In-person workshop + supervision as usual (W+SAU): 3-hour, in person workshop + 3, 1-hour group supervision sessions over 3 weeks post-training  In-person workshop + experiential supervision (W+SUP^+^): 3-hour, in person workshop + 3, 1-hour group supervision sessions over 3 weeks post-training  *Training content:*  W used didactic presentation, video examples, live modeling  by the instructor, and role-plays  SAU included rapport building, agenda setting, case narrative and conceptualization, planning for session, discussing alliance, and case management/ administrative issues  SUP^+^ used scaffolding and experimental learning strategies. Included SAU activities plus performance feedback based on recording review, modeling and role-playing with feedback | *N*=40  *Inclusion*: Students enrolled in Clinical Psychology and School-Clinical Child Psychology doctoral programs at a professional school of psychology, and students in Masters’ training programs in Social Work and Mental Health Counseling  *Exclusion*: Prior practical experience conducting CBT or extensive experience practicing CBT techniques  *Previous experience with EBP*: Inexperienced in cognitive restructuring  *Discipline*:  Social work trainee=10%  Master’s-level counselor trainee=20%  Doctoral-level counselor trainee=70%  *Demographics*:  Mean age = 24.7 years  Female = 90%  Caucasian = 67.5% | Simulated clients (acting as 12-year old girls struggling with life stressors and symptoms of depression) | *Assessment measures*:  Competence: Measured by expertise quality and global CBT competence domains from the Manual for the CBT Competence Observational Measure of Performance with Youth Depression (CBTCOMP-YD; Lau & Weisz, 2012). Expertise quality scored on 3-point Likert scale (1=novice, 3=expert) and global CBT competence scored on 10-point Likert scale (1=novice, 5=intermediate, 10=expert)  Fidelity: Measured by Therapist Integrity to Evidence Based Interventions (TIEBI; Bearman et al., 2012); simulated client interaction rated by two masked coders scored on a 0 to 4 scale, with higher scores indicating better practice fidelity (range 0-4)  Knowledge: Measured by 15-item test, total score was number of correct items (range 0-15)  *Assessment timeframe*:  Pre-training, post-training, and following each supervision meeting (3 weeks of follow-up) | *Random sequence generation*: Unclear  *Allocation concealment*: Unclear  *Masking*: Low, coders masked to condition  *Incomplete outcome data*: Low, no loss to follow-up  *Selective outcome reporting*: Low  *Overall risk of bias*: Medium |
| Beidas 2012  *Location*:  United States  *Setting*:  Community Participants, practice settings varied  *EBP evaluated*:  Cognitive Behavioral Therapy (CBT) (Coping Cat Program)  *MH condition*:  Youth Anxiety | *Training Format:*  In-person didactic workshop + virtual consultation (W+VC): In-person didactic workshop (6 hours) + virtual consultation (3 months)  In-person experiential workshop + virtual consultation: In-person didactic and experiential workshop (6 hours) + virtual consultation (3 months)  Online training + virtual consultation: Online training (6 hours) + virtual consultation (3 months)  *Training Content:*  W used PowerPoint presentation and viewing of videotapes of representative youths receiving treatment  W^+^ focused on core CBT principles via didactics and modeling, focus on active learning through role-playing and small-group discussion with guided questions  OLT: Self-paced online training resembled W condition with step-by-step instructions for each session, videos of treatment sessions, therapist tips, links to research articles  VC by telephone or virtual group meeting via computer. Content designed with participant input and included case consultation, didactics, practice with concepts, assistance in implementing treatment in practice context | *N* = 115  *Inclusion:* Work in community with children ages 8-17 with anxiety disorders, trained in mental health, read/speak English, access to a computer or telephone  *Exclusion*: ≥8 hours of prior training in CBT for child anxiety  *Previous Experience with EBP*: All had ≤8 hours of prior training in CBT for child anxiety  *Discipline*:  MD or DO = 7%  PhD or Psy.D. = 9%  EdD = 2%  Social worker = 24%  Master’s-level counselor = 41%  Graduate student = 16%  Other = 1%  *Demographics*:  Mean age = 35.9 years  Female = 90%  Caucasian = 67% | N/A – no clients in study | *Assessment measures:*  Adherence: Measured by 7-point Likert scale checklist assessing for presence of six CBT competencies for child anxiety (range 0-6), cut-off score of 70% = trained to criterion.  Competence: Measured by Likert scale (range 1-7), cut-off score of 3.5 = trained to criterion  Knowledge: Measured by 20-item test assessing knowledge of CBT for youth anxiety (range 0-20), cut-off score of 70% = trained to criterion  Satisfaction: Measured by 12-item questionnaire (items range 1 to 5) and summed  *Assessment timeframe:*  Baseline, post-training, and 3-month follow-up after virtual consultation | *Random sequence generation*: Low; unpredictable randomization sequence generated  *Allocation concealment*: Low; equal allocation concealment, random assignment occurred at the level of training date  *Maskinging*: Low; coders, simulated clients masked to participants’ condition  *Incomplete outcome data*: Low, 98% completed post-training and 87% completed follow-up  *Selective outcome reporting*: Low  *Overall risk of bias*: Low |
| Bennett-Levy 2012  *Location*:  Australia  *Setting*:  Community Participants, priority given for participants in regional, rural, and remote areas  *EBP evaluated*:  Cognitive Behavioral Therapy (CBT)  *MH condition:*  Depression, panic disorder, agoraphobia, and generalized anxiety disorder | *Training format:*  Online training (OLT): 30 modules (typically take 30-60 minutes to complete), self-paced and given access for 12 weeks  Online training + supportive calls (OLT+SC): OLT + Skype or telephone supportive calls (15 minutes delivered biweekly, or 6 times, over 12 weeks)  *Training content:*  OLT: PRAXIS program – a structured, step-by-step introductory online CBT program. Modules focused on CBT skills. Text- and video-based, and uses several illustrative case examples and quizzes. A key element is regular video demonstrations by experienced therapists.  SC: Provided by experienced clinical psychologist with extensive CBT experience. Purpose of supported training was to support participants’ progress with PRAXIS and to address any CBT or IT questions. Case supervision was not provided. | N=49  *Inclusion*: Practicing in a counselling role with ≥1 year of experience in a counselling and /or mental health role, relevant degree (psychology, social work, counselling, medicine) or completing a degree, and/or having appropriate professional qualifications (e.g., nursing), have a broadband connection, have facilities to use Skype or phone, have time and workplace support to do course  *Exclusion*: NR  *Previous experience with EBP*: Independent online training group scored significantly higher on pre-training utilization of CBT (*p*<.05)  *Discipline*:  Psychologist = 55.1%  Nurse = 8.2%  Social worker = 24.5%  Doctor = 2.0%  Counselor = 10.2%  *Demographics*:  Mean age = 45.9 years  Female = 81.6%  Race/Ethnicity = NR | N/A – no clients in study | *Assessment measures:*  Knowledge: Measured by 20-item, multiple-choice test adapted from CBT Questionnaire (Myles et al., 2003). Also measured by self-rated knowledge of 27 CBT skills on measure developed for study.  Adoption: Measured by self-report of 27 CBT skills use with last 5 clients (range 0-27) on measure developed for study  Skill acquisition: Measured by self-rating of skills-in-action on 27 CBT skills on measure developed for study  *Assessment timeframe:*  Baseline, Post-training, 4-week follow-up | *Random sequence generation*: Unclear  *Allocation concealment*: Unclear  *Masking*: Medium; participants not masked  *Incomplete outcome data*: Low, 95.9% completed follow-up  *Selective outcome reporting*: Low  *Overall risk of bias*: Medium |
| Chu 2017  *Location*: United States  *Setting*: Youth practice settings  *EBP evaluated*:  Cognitive Behavioral Therapy (CBT)  *MH condition*:  Youth anxiety | *Training format:*  Online training + online expert streaming consultation (OLT+ES): OLT (6.5 hours) + non-interactive ES  Online training + in-person peer consultation (OLT+FS): OLT (6.5 hours) + Fact sheet (FS)  Online training + fact sheet self-study (OLT+PC): Online workshop (6.5 hours) + PC (10, 1-hour meetings over 10 weeks)  *Training content:*  OLT: Group viewing of online workshop of expert teaching CBT for anxious youth (diagnostic assessment, key CBT interventions).  ES: Participants accessed weekly video of an expert providing deidentified supervision to on-site trainees on their youth anxiety cases. Participants did not interact with the expert or trainees. Each video was available online for 1 week.  FS: Participants emailed brief clinical resource sheets (one to two pages each) weekly and reviewed at own pace across 10-week period.  PC: Participants met in person weekly for peer-led groups across a 10-week period to discuss CBT in relation to their current anxiety caseloads. To facilitate discussion, participants were provided the FS fact sheets and a prompt sheet. Study staff did not provide feedback or facilitate but attendance was taken. | *N* =35  *Inclusion*: Enrollment in/ graduation from a master’s or doctoral program in social work, counseling, clinical, or school psychology;  need to be actively working in youth practice settings.  *Exclusion*: Prior formal training in CBT for youth anxiety;  refusal to participate in workshop  *Previous Experience with EBP*:  No previous training for CBT related to youth anxiety  *Discipline*:  Ph.D. = 9%  Master’s = 31%  Ed.S. = 3%  Student = 57%  *Demographics*:  Mean age = NR  Female = 89%  Caucasian, non-Hispanic = 69% | N/A – no clients in study | *Assessment measures:*  Adoption: Measured by self-report of specific CBT skills for youth anxiety cases. Adapted from prior therapist-reported CBT use measures (Kuriyan & Pelham, 2012). Responses rated from 0 (no clients) to 3 (all clients) and averaged.  Skill acquisition: Measured by single self-report item of enhanced skill/ability on 0 to 8 Likert scale.  Knowledge: Measured by four versions of 20 to 22 item multiple-choice test adapted from Society for Clinical Child and Adolescent Psychology continuing education exams.  Satisfaction: Measured by 7-items on 1 to 5 Likert scale (conditions for ES, FS, and PC). Mean score computed for scale.  *Assessment timeframe:*  Pre-training, Mid-point (Week 5), Post-training (Week 10) | *Random sequence generation*: High; two participants re-randomized due to requirements for condition  *Allocation concealment***:** Unclear  *Masking*: Unclear  *Incomplete outcome data*: Low; 91% completed post-training assessment  *Selective reporting*: Low    *Overall risk of bias:* Medium |
| Cohen 2016  *Location*:  United States  *Setting*:  Residential treatment facilities (RTF) serving adjudicated youth  *EBP evaluated*: Trauma Focused Cognitive Behavioral Therapy (TF-CBT)  *MH condition*:  Posttraumatic Stress Disorder (PTSD) | *Training format:*  Online training + online consultation course (OLT+OC): TF-CBT*Web* with access to free online consultation course as needed (time: 10 hours)  Online training + in person workshop + phone consultation (OLT+W+PhC): TF-CBT*Web* + in-person workshop (time: 2 days) + 12 months of bi-monthly consultation calls with expert (time: NR).  *Training content:*  OLT: TF-CBT*Web* which is self-paced and includes modules on step-by-step instructions, demonstration videos, clinical challenges, cultural considerations.  PhC: Consultation calls included behavioral rehearsal for specific TF-CBT skills, positive feedback for specific actions indicating model fidelity, and feedback on cases. | *N*=129  *Inclusion*:  RTF programs serving adjudicated youth aged 12-17 years; employing two or more mental health therapist who provided ongoing treatment to youth in the RTF; RFT leadership agreed to participate and appropriately protected human subjects.  *Exclusion*: NR  *Previous experience with EBP*: No RTFs were currently implementing an evidence-based trauma-focused therapy and none had received formal trauma-informed training prior to start of project.  *Discipline*:  Licensed social worker=24.8%  Licensed mental health counselor=21.7%  Other licensed therapists=8.6%  Unlicensed therapists=45.0%  *Demographics*:  Mean age = 39.3 years  Female = 80.7%  Caucasian = 90.0% | *N*=81  Inclusion: Adjudicated youth aged 12-17 who were residing at participating RTF programs, agreed to trauma screening by assigned therapist; reported ≥1 traumatic experience and scored ≥22 (moderately severe PTSD) on UCLA PTSD Reaction Index; would continue to be treated by study therapist.  Mean age = 15.1 years  Female = 52.8%  Caucasian = 62.1% | *Assessment measures:*  Adherence: Measured by 9-item self-report checklist completed by therapist immediately after treatment session. Therapist indicated which components were delivered during the session. A composite score ranging from 0 to 2 was derived for each case. Composite score derived by a) therapist providing 8-30 sessions of TF-CBT (1 point) and all practice components delivered in appropriate order (1 point). A rating of 2 indicated meeting required adherence standard.  Knowledge: Measured by 40-item test. Each item has a single correct answer and the score indicated total numbers of correct answers (range 0 to 40).  Client outcomes measured by:  1) Therapy completion rate: proportion of clients who completed treatment.  2) PTSD symptoms measured by 22-item, self-report UCLA PTSD Reaction Index, Adolescent Version (total score range 0-88).  3) Depression symptoms were measured by 13-item self-report Mood and Feelings Questionnaire - short version (total score range 0-26)  *Assessment timeframe:*  Pre-treatment, weekly therapist self-report during treatment, post-treatment (between 8 and 30 sessions). | *Random sequence generation*: Low, randomized using sealed envelopes  *Allocation concealment*: Low, used sealed envelopes  *Masking*: Low, clients and research coordinator masked to therapist condition  *Incomplete outcome data*: High, 51.2% of therapists completed study; 39.5% of clients completed study  *Selective outcome reporting*: Low  *Overall risk of bias*: Medium |
| Cooper 2017  *Location*:  United States & Canada  *Setting*:  Community participants, practice settings varied  *EBP evaluated*: Cognitive Behavioral Therapy for eating disorders (CBT-E)  *MH condition*:  Eating Disorders | *Training format:*  Online training (OLT): 9 hours (with 20 weeks to complete) + access to online library of resources on CBT-E  Online training + supportive calls (OLT+SC):  OLT (9 hours with 20 weeks to complete) + 12 telephone calls, ≤30 minutes (weekly for first 4 weeks, then biweekly).  *Training content:*  OLT: Linear in nature, expert gives practical description of how to implement CBT-E through multiple brief video presentations interspersed with handouts, formative learning exercises, video recordings of acted illustrations of CBT-E, knowledge tests. Online library also had supplemental material on working with specific client subgroups.  SC: Calls were purely supportive in nature (to encourage completion of online training and implement CBT-E with their training cases). | *N*=156  *Inclusion*:  Licensed mental health professionals; have previous training in delivering short-term psychological treatments; work with individuals with eating disorders; be willing to be randomized and partake in web-centered training, be willing to devote at least 9 hours to training program; be able to treat 1 or more clients using CBT-E during the 20-week period of training; and provide informed, digital consent  *Exclusion*: NR  *Previous experience with EBP*: NR  *Discipline*:  Clinical Psychologists = 41.0%)  Social Workers = 28.9%  Other (counseling, family therapy, psychiatry, psychiatric nursing) = 30.1%  -Median years of FTE clinical experience. = 5.9 (IQR 3 – 13.3; range 0-36 years)  *Demographics*:  Median age = 36 years  Female = 93.3%  Caucasian = NR | N/A – no clients in study | *Assessment measures*:  Knowledge: Measured by 22-item measure of knowledge and understanding of CBT-E and its implementation (Cooper et al., 2015). Items were summed for total score (range 0-22).  *Assessment timeframe:*  Pre-training, post-training (20 weeks), and 6-month follow-up post-training. | *Random sequence generation*: Low, used minimization  *Allocation concealment:* Unclear  *Masking:* Unclear  *Incomplete outcome data:* High; 71.3% completed post-training assessment and 48.8% completed follow-up assessment  *Selective reporting:* Low  *Overall risk of bias:* Medium |
| Dimeff 2009  *Location*:  United States  *Setting*:  Practice settings varied  *EBP evaluated*:  Dialectical Behavior Therapy (DBT) skills  *MH Condition:*  Borderline Personality Disorder and/or substance abuse | *Training format:*  Treatment Manual (TM): treatment manual (Linehan, 1993) with suggested study guide (time NR)  Online training (OLT): Self-paced OLT (estimated time of 20 hours)  In-person workshop (W): 2-day workshop with expert DBT trainers  *Training content:*  TM: Written DBT skills treatment manual (Linehan, 1993) along with suggested study guide  OLT: Five modules on DBT skills. Included practice exercises, knowledge checks, and clinical simulations of a fictional DBT skills group.  W: DBT experts generated and revised workshop materials. Participants received binder with training PowerPoint slides. | *N* = 150  *Inclusion*:  DBT naïve (defined as never received formal training or read either of the DBT manuals or participated on a DBT consultation tea; access to computer with sound card and internet; currently treating at least 1 individual how had substance abuse problems and/or chronic suicidality (students were exempt from this criterion); availability to attend the workshop if assigned to condition.  *Previous Experience with EBP*:  DBT Naïve  *Discipline*:  Psychiatrist = 1.3%  Psychologist = 22.8%  Chemical Dependency Counselor = 2.7%  Social Worker (MSW) = 22.8%  Bachelor’s Level Counselor =12.1%  Other = 16.8%  *Demographics*:  Mean age = 39.6 years  Female= 69.8%  Caucasian= 80.7% | Simulated clients (representative of typical borderline personality disorder client needing help following a distressing event) | *Assessment measures*:  Adherence: Measured by performance-Based Role Play (PBRP), a 15-minute recorded phone call with simulated client and coded by rater. Measured presence/absence of 65 DBT skills. Incorrect use downgraded and categories weighted with more points for more thorough skills training. Each of the 65 possible DBT skills had maximum score of 9.  Competence: Measured by PBRP (see Adherence). Scored on 6-point Likert rating from 0 (poor) to 5 (competent).  Adoption: Measured by participants reported the number of times they taught or applied specific DBT skills in clinical work in the past 30 days rated on scale from 0 (0 times) to 6 (≥20 times)  Knowledge: Measured by 82-item multiple choice test developed by DBT experts. Score is proportion of items correct  Satisfaction: Measured by 14-items on 1 to 5 Likert scale at post-training  *Assessment timeframe:*  Pre-training, post-training, 90-day follow-up | *Random sequence generation*: Low; used minimization  *Allocation concealment*: Unclear  *Masking*: Medium; simulated clients masked but unclear if raters masked  *Incomplete outcome data*: Medium (88% completed post-training and 70% completed 90-day follow-up)  *Selective reporting*: Low    *Overall risk of bias*: Medium |
| Dimeff 2011  *Location*:  United States  *Setting*:  Practice settings varied  *EBP* e*valuated*:  Dialectical Behavior Therapy (DBT) (distress tolerance skills)  *MH condition:*  Borderline Personality Disorder and substance abuse | *Training format:*  Treatment manual (TM): training manual; self-study (Time 2.5 hours).  Online training (OLT): Multimedia, interactive e-Learning course covering same content as Manual group (time: 2.5 hours)  Placebo online training (pOLT): online simulation (time: 2.5 hours)  *Training content:*  Manual: Participants given a copy of DBT Crisis Survival Strategies portion of the Distress Tolerance module of the Skills Training Manual for self-study. Materials included overview of module, instructions and discussion points forteaching skills, and client handouts and homework sheets to facilitate skills acquisition and strengthening  OLT: Course had 5 modules and contained expert insights, practice exercises, and knowledge checks. Users could also download/print session transcripts, note taking guide, and glossary  pOLT: Online simulation of treatment for client with borderline personality disorder in an inpatient setting and presented on behaviors common to clients with disorder | *N*=132  *Inclusion*: Mental Health Participants, drug treatment Participants, or students in training programs to become treatment Participants; currently treating at least one client with substance abuse problems and/or was chronically suicidal (students were exempt from this criterion); and had limited exposure to DBT  *Exclusion*: Previous reading of any portion of either two DBT manuals, were on a DBT consultation team, had attended a DBT intensive training or a 2-day skills training workshop, or knew how to implement DBT distress tolerance skills.  *Previous* *Experience with EBP*:  DBT Naïve  *Discipline*:  Psychologist = 1.5%  Psychiatric Nurse = 0.8%  Psychiatric Nurse Prac. = 1.5%  Chemical Dependency Counselor = 12.1%  Social Worker (MSW) = 12.1%  MH Counselor/Therapist =41.7%  MH Counselor/Technician= 17.4%  Other = 12.9%  *Demographics*:  Mean age = 43.8 years  Caucasian= 82.6%  Female= 74.2% | N/A – no clients in study | *Assessment measures*:  Adoption: Measured by self-report of number of times taught or applied a specific new skill learned in program to clinical work.  Knowledge: Measured by 23-item multiple choice test on DBT Distress Tolerance Skills. Score was proportion of items correct minus any missing items.  Satisfaction: Measured by a six-item Usability subscale rated on 7-point Likert scale from 1 (very boring/difficult/not at all) to 7 (very interesting/easy/informative) and a  7-item Acceptability subscale rated  on a 5-point Likert scale from 1 (not  enjoyable/helpful/relevant/likely) or (didn’t hold my interest) to 5 (extremely enjoyable/helpful/relevant/  likely) or (completely held my interest). Items averaged for each subscale.  *Assessment timeframe:*  Pre-training, post-training, 2-week, 7-week, 11-week, and 15-week follow-ups | *Random sequence generation*: Low; used minimization    *Allocation concealment*: Unclear  *Masking*: Low; research staff masked to participant condition  *Incomplete outcome data*: Medium; up to 16.7% of participants did not complete follow-up assessments  *Selective reporting*: Low  *Overall risk of bias*: Low |
| Dimeff 2015  *Location*:  United States  *Setting*:  Community participants, practice settings varied  *EBP evaluated*:  Dialectical Behavior Therapy (DBT) (chain analysis and validation strategies)  *MH condition*:  Borderline Personality Disorder with and without substance abuse problems | *Training format:*  Online Training (OLT): Self -paced, covered same content at W (time: 12 hours total)  In-person workshop (W):  (time: 12 hours over 2 days)  Treatment manual (TM): self-paced study (time: NR)  *Training content:*  OLT: access to two online training courses which incorporated expert commentaries, practice exercises, simulated therapy, printable handouts and study guides, and knowledge checks.  W: Included both passive learning (e.g., lecture, video) and active learning methods (e.g., role-play, experiential exercises). Approximately two-thirds of the ILT was devoted to DBT chain analysis and one- third to DBT validation strategies. All participants attended the same, one-time workshop. Participants given binder of printed PowerPoint slides to capture their notes during the workshop.  TM: Training materials on DBT chain analysis (133 pages) and DBT validation strategies (59 pages). The manual was created specifically for this trial and contained expert descriptions and explanations of each concept, clinical scenarios, and practice examples and was accompanied by a suggested study guide. | *N* = 172  *Inclusion*: minimal previous experience using DBT strategies in training; had not read DBT manuals or received 3 or more days of DBT training; access to a computer with a sound card and internet; treating at least one individual with substance abuse problems; available to participate in workshop if assigned to condition  *Exclusion*: NR  *Previous experience with EBP*:  Naïve to DBT (unfamiliar with DBT manuals or received ≤3 days of training)  *Discipline*:  Psychiatrist = 0.6%  Psychologist = 3.5%  Psychiatric nurse = 1.2%  Social worker = 16.3%  Chemical Dependency Counselor = 21.5%  Master’s-level counselor = 20.9%  Bachelor’s-level counselor = 3.5%  Student = 8.1%  Other = 24.4%  *Demographics*:  Mean age = 39.8 years  Female = 76.2%  Caucasian = 78.5% | N/A – no clients in study | *Assessment measures*:  Competence: Measured by three 20-minute role plays with feedback by experts, global proficiency score computed (range 0 to 3)  Adoption: Measured by self-reported frequency of clinical strategies, items rated on 6-point scale: 1 (0 times), 2 (1-4 times), 4 (5-9 times), 5 (15-20 times), and 6 (>20 times)  Knowledge: Measured by 55-item chain analysis test and 41-item validation test, mean score for proportion of items answered correctly and total combined score calculated  Satisfaction: Measured by 9-item generic scale (range 1-5), averaged for total score  *Assessment timeframe:*  Baseline, post-training, 30-, 60-, and 90-dayfollow ups | *Random sequence generation*: Low; used minimization  *Allocation concealment*: Unclear  *Masking*: Low; simulated clients masked to participants’ condition  *Incomplete outcome data*: High; 86% completed baseline, 73% completed post-training assessment  *Selective outcome reporting*: Low  *Overall risk of bias*: Medium |
| Fu 2015  *Location*:  United States  *Setting*: VA Medical Centers  *EBP evaluated*:  Motivational Interviewing (MI)  *MH condition*:  Smoking | *Training format:*  In-person workshop (W): In-person, half day MI workshop + self-study materials (time: half day)  In-person workshop + booster sessions + peer coaching (W+BS+C): In-person, half day MI workshop + self-study materials + six, 1-hour booster sessions with coaching and practice with simulated client (time: half day + 6 hours over 12 weeks)  *Training content:*  W: Expert trainers covered the foundations of MI and situations for use, and favored interactive over didactic teaching methods. Participants watched video examples of MI techniques and had the opportunity to practice MI skills. Additionally, a tobacco cessation expert presented on the national guideline recommendations to address tobacco use and how to incorporate MI with guidelines.  Self-study: Participants given copies of the PowerPoint presentations, tobacco cessation client case studies, MI work- sheets, and MI pocket cards developed by the VA National Center for Health Promotion and Disease Prevention.  BS+C: Three group sessions and three 1:1 interactions with simulated clients facilitated by MI champions. For the three small group booster sessions, champions had detailed agenda of suggested activities focusing on specific MI skills, printed client cases, and exercises to use with suggested time parameters. One-on-one coaching booster sessions focused on skills practice using a simulated client. The participant was connected to the simulated client via telephone for up to 30 min, first in role-play and then with the simulated client providing feedback to the participant. An MI champion listened to the entire dialog and provided additional feedback. | *N* = 34  *Inclusion*: Any primary care clinician (physician, physician assistant, nurse practitioners, nurses, pharmacists)  *Exclusion*: Trainees  *Previous experience with EBP*: 44.1%  Previous tobacco cessation counseling experience: 29.4%  Previous training on client communication: 58.8%  *Discipline*:  Primary care participant = 29.4%  Nursing = 50.0%  Pharmacy = 20.6%  *Demographics*:  Mean age = 41.3 years  Female = 79.4%  Caucasian = 70.6% | Simulated clients (actor, portraying a client who wanted to stop smoking) | *Assessment measures*:  Adherence: Measured by objective structured clinical examinations (OSCE) during interaction with 2 simulated clients; rated by single, masked rater using MITI; percent MI adherent is total # of MI adherent behaviors during client encounter divided by total number of behaviors (adherent + non-adherent)  Competence: Measured by Global MI spirit rating on 5 point Likert scale, with higher score indicating more MI-consistent  Knowledge: Measured by 10-item, multiple-choice scenario-driven questionnaire; score is % of questions correct  *Assessment timeframe:*  Baseline and 3-month FOLLOW-UP post-initial training | *Random sequence generation*: Low; used statistical computer algorithm  *Allocation concealment*: Unclear  *Masking*: low, rater masked to participants’ conditions  *Incomplete outcome data*: Medium; 94.1% completed pre and post-assessments, 79.4% completed pre- and post-clinical examinations  *Selective outcome reporting*: Low  *Overall risk of bias*: Low |
| Gega 2007  *Location*:  United Kingdom  *Setting*: University nursing program  *EBP evaluated*: Exposure Therapy (ET)  *MH condition*:  Phobia and Panic disorders | *Training format:*  In-person workshop (W): In-person, group lecture from qualified nurse behavior therapist. PowerPoint presentation (Time: 1 hour)  Online training (OLT): FearFighter classroom setting with participants on individual computer terminals. (Time: 1 hour)  *Training content:*  W: PowerPoint presentation with content similar to FearFighter, lecturer also answered questions and discussed issues with students.  OLT: Fearfighter described how exposure therapy works for phobia/panic, how to devise personalized exposure step-by-step, and how to troubleshoot difficulties (Time: 1 hour)  *Study employed crossover design; only comparisons of initial training methods are summarized. | N = 92  *Inclusion*: Mental health nursing students  *Exclusion*: NR  *Previous experience with EBP*:  No prior training in exposure therapy  *Discipline*:  Mental health nursing students = 100%  *Demographics*:  Mean age = NR  Female = NR  Caucasian = NR  *English was second language for Three fourths of participants | N/A– no clients in study | *Assessment measures:*  Knowledge: Measured by two 10-item multiple choice questionnaires. Each question could have 0 to 4 true answers, for a total minimum score of 0 and maximum score of 80 for the two questionnaires.  Satisfaction: Measured by student rating of (a) how much they enjoyed the session, (b) how useful they found it, range 0 (not at all) to 8 (extremely), and (c) whether they would like to learn more about exposure and related therapy, range 0 (not really) to 8 (very much more)  Skill acquisition: Measured by answers to 5 questions for each of two case scenarios (individual with spider-phobia and individual with agoraphobia/panic). Each question was rated from 0 (no or irrelevant answer) to 8 (accurate and complete answer) and averaged.  *Assessment timeframe:*  Pre-training, Post-training | *Random sequence generation*: Low; randomization via computer-generated tables, stratified by student’s pathway of study  *Allocation concealment*: Unclear  *Masking*: Low; case scenario answers scored by masked rater  *Incomplete outcome data*: Low; 91.3% completed all assessments  *Selective outcome reporting*: Low  *Overall risk of bias*: Low |
| Harned 2011  *Location*:  United States  *Setting*:  Community participants, practice settings varied  *EBP evaluated*: Exposure Therapy (ET)  *MH condition*: Anxiety disorders | *Training format:*  Online training (OLT): Self-guided online training of ET (time: approximately 2 hours)  Online training + motivational interviewing (OLT^+^): OLT described above + MI-based phone calls pre- and post-training (time: 1 to 2 calls, each for 20 minutes)  Placebo online training (pOLT): Self-guided online training of an unrelated therapy (time: approximately 2 hours)  *Training content:*  OLT: Self-guided OLT including theoretical foundations and step-by-step ET techniques, interactive exercises with clinical simulation with a fictional anxiety disorder client, and option to design own ET treatment, resources section, and videos of experts discussing clinical cases and addressing concerns related to therapist anxiety about ET. Each module included interactive exercises, knowledge checks to assess learner understanding, and content to address myths about ET.  OLT^+^: OLT described above plus 1 to 2 semi-structured interviews based on MI principles and strategies to decrease ambivalence about adopting ETs due to attitudinal barriers.  Placebo online training (pOLT): Self-guided OLT of unrelated therapy (DBT treatment strategies). Comparable in quality, length, and design to the OLT and contained no overlapping content. | *N=*46  *Inclusion*:  ≥18 years of age, currently employed as a treatment participant at a mental health agency or students working to obtain a professional degree in mental health-related field, currently treating clients with anxiety disorders or engaged in educational curriculum with training in anxiety disorders, access to a computer with a sound card and internet connection, and minimal prior exposure to exposure therapies.  *Exclusion*:  Read any portion of published treatment manuals on an empirically-supported ET, attended a lecture, workshop, or intensive training on ET.  *Previous experience with EBP*:  Naïve or minimal experience in exposure therapy  *Discipline*:  Psychologist = 23.9%  RN/ARNP = 4.3%  Social worker = 26.1%  Master’s level counselor= 26.1%  Bachelor’s level participant = 4.3%  Other = 15.2%  *Demographics*:  Mean age = 41.4 years  Female = 82.6%  Caucasian = 73.9% | N/A– no clients in study | *Assessment measures:*  Adoption: Measured by four self-report items assessed participants’ application of course content in clinical practice. Items were averaged for analyses.  Knowledge: Measured by 27-item multiple choice test (with 4-5 response choices per item) assessing knowledge of course content and ability to apply knowledge in hypothetical clinical scenarios. Score was proportion of items answered correct  Satisfaction: Measured by self-report of satisfaction with assigned training condition. Included a7-item acceptability subscale, which assessed course relevance using 5-point Likert scale from 1 (not enjoyable) to 5 (extremely enjoyable) and 5-item usability subscale assessed usability of training using 7 point Likert scale from 1 (very boring) to 7 (very interesting). Subscale scores were created by averaging items.  *Assessment timeframe*:  Pre-training, post-training, 1-week follow-up | *Random sequence generation*: Low; minimization used  *Allocation concealment*: Unclear  *Masking*: N/A – no masked assessments  *Incomplete Outcome Data*: Low; 100% completed at least one measure at post-training and 1-week follow-up  *Selective outcome reporting*: Low  *Overall risk of bias*: Medium |
| Harned 2014  *Location*:  United States  *Setting*: Practice settings varied  *EBP evaluated*: Exposure Therapy (ET)  *MH condition*: Anxiety Disorders | *Training format:*  Online training (OLT): self-paced online course (time: 10 hours)  Online training + motivational enhancement (OLT^+^): OLT above + two brief, online ME interventions (first ME intervention 5 minutes, second time NR)  Online training + motivational enhancement + learning community (OLT^+^+LC): 8, 1-hour meetings over 12 weeks)  *Training content:*  OLT: Participants could choose to review structured didactic materials and/or learn experientially by engaging in simulated clinical scenarios with virtual clients. OLT also contained videos of exposure experts, example treatment forms, and a list of treatment manuals. Experiential learning occurred via 35 simulated clinical scenarios in which learners treated six virtual anxiety disorder clients and received feedback on their performance. To finish the course, learners had to successfully complete the most difficult scenario.  ME: Two brief interventions aimed at addressing potential attitudinal barriers to learning and using ET: A brief (5-minute) video that played when participants first accessed the OLT. The second intervention occurred immediately upon completing the OLT and was an additional online module involving a simulated conversation with a “virtual ET consultant” that incorporated a variety of strategies derived from MI.  LC: Participants could call in via telephone or use computer video conferencing. LC was facilitated by experienced ET clinician using a structured curriculum. The first five meetings targeted knowledge acquisition and practice and included discussing assigned content from OLT and engaging in role-plays, active practice of core concepts. Participants were given weekly assignments to complete specific sections of the OLT and practice ET elements. Final three meetings targeted increasing use of and clinical proficiency in ET in clinical practice. | *N* = 181  *Inclusion*:  Currently working as mental health participant/student; currently providing individual therapy to ≥ 3 clients w/ anxiety disorder and planning to continue for duration of study; access to technology required for study training; able to commit to study time requirements; Bachelor’s degree or higher  *Exclusion*:  More than minimal prior exposure to ET defined as (a) clinical experience using ET for ≥8 sessions or received clinical supervision on ET (b) training focused on ET, (c) previously viewed any of OLT used in study or two other OLTS of an exposure-based treatment developed by workgroup, (d) read > half of any ET treatment manual within last 3 years  *Previous experience with EBP*:  No more than minimal prior exposure to ET  *Discipline*:  Psychiatrist = 3.5%  Psychologist = 14.0%  Psychiatric nurse = 1.8%  Social worker = 27.5%  Master’s-level counselor = 28.7%  Bachelor’s-level counselor = 1.8%  Student = 22.8%  *Demographics*:  Mean age = 37.4 years  Female = 71.3%  Caucasian = 72.1% | N/A – no clients in study | *Assessment measures:*  Competence: Measured by observer-rated proficiency based on three 20-min simulated role plays, global clinical proficiency score computed (range 0=no proficiency to 5=excellent proficiency)  Adoption: Measured by self-reported use of nine ET procedures per anxiety disorder client.  Knowledge: Measured by 49-item, multiple-choice instrument developed for study to assess knowledge of course content/ability to apply in hypothetical clinical scenarios, scored on proportion of items correct  Satisfaction: Measured by OLT 16-item adapted version of the E-Learner Satisfaction Survey (ELS; Wang, 2003; range 16-112). Separate 9 -item questionnaire used for ME and LC components, rated on 5-point Likert scale  *Assessment timeline:*  Baseline, post-training, 6- and 12-week follow up | *Random sequence generation*: Low; minimization used  *Allocation concealment*: Unclear  *Masking*: Low; coders, simulated clients masked to participants’ condition  *Incomplete outcome data*: High; 80.1% completed post-training; 76.2% completed 12-week follow-up  *Selective outcome reporting*: Low  *Overall risk of bias*: Medium |
| Henggeler 2008  *Location:*  United States  *Setting*: Practice settings varied  *EBP evaluated*: Contingency Management (CM) within Multisystemic Therapy (MST)  *MH condition*: Marijuana abuse | *Training format:*  In-person workshop (W): in-person training (time: 2 days)  In-person workshop + Intensive Quality Assurance (W+ IQA): Workshop + $150 for implementing CM voucher system + supervision in CM + weekly expert case consultation on CM cases (time: 6 months).  *Training content:*  W: Participants asked to read manual prior to workshop which included didactics, expert demonstration, small-group exercises, and role plays with reinforcement and corrective feedback + $150 to facilitate any aspect of MST not limited to CM + phone access to CM expert for consultation if desired (6 months).  IQA: CM skills/competency incorporated into clinician development plans, and quarterly booster training focused on therapist practice and role-play of CM components. | *N=30*  *Inclusion*:  Community practitioners previously trained in MST  *Exclusion*: NR  *Previous experience with EBP*: NR  *Discipline*:  Bachelors level therapist =17%  Masters level therapist= 83%  *Demographics*:  Mean age = 33 years*  Female = 83%  Caucasian = 57%  *Therapists in W condition were significantly older those in W+IQA condition. | *N*=70 youths (and their caregivers)  Inclusion: Families in MST; youth meet diagnostic criteria for marijuana abuse or dependence based on structured clinical Interview for DSM-IV  Youth Demographics:  Mean age = 15 years  Female = 23%  Caucasian = 36%  Previous MH or substance use treatment = 56%  Caregiver Demographics:  Mean age = 40.8  Female = 90%  Median education level= High school graduate | *Assessment measures:*  Adherence: Measured by youth and caregiver rating of therapist behavior using a 9-item CM Therapist Adherence Measure. Scale included 5 items on 4-point scale on cognitive-behavioral aspect of CM rated from 1 (low) to 4 (high) and 4 items on CM monitoring aspects on 3-point scale from 1 (low) to 3 (high).  *Assessment timeframe*:  Pre-training, 1, 2, 3, and 4 months post-training | *Random sequence generation*: Unclear  *Allocation concealment*: Unclear  *Masking*: Unclear if youths and caregivers masked to therapists’ condition  *Incomplete outcome data*: Unclear; did not report on attrition/loss to follow-up  *Selective outcome reporting*: High; did not report on sustainability period  *Overall risk of bias*: Medium |
| Hubley 2015  *Location:*  United States  *Setting*:  Practice settings varied  *EBP evaluated*: Behavioral Activation (BA)  *MH condition*: Depression | *Training format:*  Online training (OLT): Self-paced (time: 90-120 minutes)  Online placebo control training (pOLT): pOLT (time: 90-120 minutes)  *Training content:*  OLT: Six modules on BA including treatment overview, rationale, identifying intervention targets, and developing action plan.  pOLT: Online training in other therapy (Dialectical Behavior Therapy validation strategies) | *N*=49  *Inclusion*:  Aged ≥18 years, currently employed at community mental health agencies or were students working to obtain a professional degree in a mental-health related field.  *Exclusion*:  Exposure to any portion of previously published treatment manuals for BA or attendance at a lecture, workshop, or other intensive training on BA.  *Previous experience with EBP*: Minimal exposure to BA  *Discipline*:  Psychiatrist =2.2%  Psychologist =52.2%  Psychiatric nurse practitioner =2.2%  Social worker (MSW) =21.7%  MH counselor/therapist =15.2%  Student=2.2%  Other =4.3%  *Demographics*:  Mean age = 45.6 years  Female = 80.4%  Caucasian = 78.3% | N/A – no clients in study | *Satisfaction*: Measured by 13-item assessment that examined course relevance and usability (BA online training course only). Course relevance measured with mean of 8-items on a 5-point Likert scale and usability was measured with mean of 5-items on a 7-point Likert scale. Higher scores indicated greater usability/relevance. Used cut-offs for adequate relevance (3.5) and usability (5.0).  *Knowledge*: Measured by 13-item, multiple choice BA Knowledge Test (BAKT) and the BA Recall Test (BART), a spontaneous recall memory test in which participants were asked to name as many BA concepts and techniques as they could recall from the online training. The score was the number of correct responses; participants were not penalized for incorrect responses. Coders masked to training condition awarded one point per acceptable response.  *Assessment timeframe*:  Pre-training, post-training, 1 week follow-up | *Random sequence Generation*: Low; minimization used  *Allocation concealment*: Unclear  *Masking*: Low; raters masked to participants’ condition  *Incomplete outcome data*: Low; 91% completed post-training and 1-week follow-up assessment  *Selective outcome reporting:* Low  *Overall risk of bias*: Low |
| Larson 2013  *Location*:  United States  *Setting*:  Addiction treatment programs  *EBP evaluated*: Cognitive Behavioral Therapy (CBT)  *MH condition*: Substance Abuse | *Training format:*  Online training + supervision (OLT+S): OLT (time: given 8 weeks) + monthly group supervisor phone calls (time: NR).  Training manual + supervision (TM+S): training manual (time: given 8 weeks) + supervision (time NR).  *Training content:*  OLT+S: Training included definition of CBT session structure, collaborate session agendas, out-of-session assignments; MI topics; functional analysis and cognitive behavioral model of addiction; skills practice, etc. OLT had 30 screens, half providing optional content, online exercise, or single questions with feedback. Each module had audio-vignette dialogue of role-play or expert commentary + monthly group supervisor phone calls on self-monitoring of CBT skill application.  TM: Same content as above in written National Institute on Drug Abuse training manual (time: given 8 weeks) + supervision as described above (time: NR). | *N* = 127  *Inclusion*:  Master’s counseling degree or bachelor’s and addiction certification; >2 years of experience; >6 clients on caseload; submission of baseline audio-tapes.  *Exclusion*:  Prior CBT training of >8 hrs. with supervised practice  *Previous Experience with EBP*: Minimal CBT experience  *Discipline*:  Certified addiction counselor: 29.9%  Registered: 15.7%  Licensed: 1.6%  None of the above: 51.9%  *Demographics*:  Mean Age = 43.9 years  Female= 66.1%  Caucasian= 76.3% | Clients were from one of the following treatment settings: standard outpatient, residential, methadone maintenance; acute inpatient, or combined modalities. | *Assessment measures:*  Adherence: Measured by audits of two pre-training tapes of client sessions submitted by counselors and three tapes post-training. One tape was randomly selected for each counselor at pre- and post-training and rated on core CBT skills on overall CBT skills, two and subdomains (generic CBT skills, and specific CBT interventions). Raters rated each multi-dimensional skill on 7-point Likert scale from 1 (not performed) to 7 (considerable use and very good application). Mean score for scales obtained. A score of 4 = “low pass” or minimally adequate application and 5 = “pass” or sufficient use.  *Assessment timeframe*:  Pre-training, post-training (8 weeks later). | *Random sequence generation*: Unclear  *Allocation concealment*: Unclear  *Masking*: Unclear if raters were masked to participant condition  *Incomplete outcome data*: Medium; 85.8% of participants provided post-training data  *Selective outcome reporting*: Low  *Overall risk of bias*: Medium |
| Martino 2011  *Location*:  United States  *Setting*:  Community addiction treatment programs  *EBP evaluated*: Motivational Interviewing (MI)  *MH condition*:  Substance abuse | *Training format:*  In person workshop by expert + consultation (EW+C): 15-hour workshop, audiotaped sessions, and 3 monthly individual face-to-face supervision sessions.  In person workshop by trainee + consultation (TW+C): two clinicians underwent training to become MI trainers and supervisors; completed 15-hr workshop to conduct training and rating-based supervision. Received monthly consultation calls from MI expert.  Treatment manual + didactic materials (TM^+^): Textbook, training videotapes, and a treatment manual; asked to spend 20 hours over 12 weeks reviewing materials.  *Training content:*  EW: Workshop provided by expert per Motivational Interviewing Network of Trainers recommendations.  TW: Same workshop as expert provided by program clinicians who became MI trainers. They all worked full- time, had master’s degrees or higher and were licensed professionals. Trainers had to meet adequate MI performance standards consistent with those used to certify. They completed another 15-hour workshop in which they learned how to conduct the MI workshop and rating-based supervision. A manual guided their preparation.  C: Provided by expert or clinician trained as MI trainer. Participants had three monthly individual face-to-face supervision session, which involved receiving rating-based feedback about their use of MI strategies and role-play practice.  TM^+^: MI textbook, training videotapes, and a treatment manual. | *N* = 92  *Inclusion* (programs):  Licensed addiction treatment facility; had not implemented MI workshop or supervision training previously  *Inclusion* (clinicians):  Employed at least 20 hours per week, treat English-speaking substance-using clients  *Exclusion* (clinicians):  Received recent formal MI workshop training or supervision  *Previous experience with EBP*:  0.9 mean hours in past year (11.9% attended lecture/presentation, 8.7% attended workshop; 2.2% received supervision; 13% use MI manual; 7.6% read MI textbook)  *Discipline*:  Alcohol/drug counseling = 70.6%  Social Work = 28.2%  Marriage/Family Counseling = 4.3%  Nursing = 1.1%  Psychology = 1.1%  Internal Medicine = 1.1%  *Demographics*:  Mean age = 44.8 years  Female = 64.7% (significantly lower % female in TT group)  Caucasian = 82.7% | Standardized client: Project staff member with previous experience as client-actor portraying client presenting as ambivalent about stopping marijuana and alcohol use.  Clients: Selected by clinicians based on clinical judgment of suitability of substituting MI for their standard practice and client’s willingness to be audiotaped. | *Assessment measures:*  Adherence: Measured by 15-item Independent Tape Rater Scale (ITRS), rating from 1 (not at all) to 7 (extensively); scores averaged to obtain mean. ITRS rating based on 40-minuted videotaped standardized role-played session with client-actor and client.  Competence: Measured by 15-item Independent Tape Rater Scale (ITRS), rating from 1 (very poor) to 7 (excellent); scores averaged to obtain mean. ITRS rating based on 40-minuted videotaped standardized role-played session with client-actor and client.  *Assessment timeframe*:  Baseline, post-workshop, post-supervision, and 12-week follow-up | *Random sequence generation*: Unclear  *Allocation concealment*: Unclear  *Masking*: Low; raters masked to participants’ condition  *Incomplete outcome data*: Medium; 90% completed post-supervision assessment, 82% completed 12-week follow-up  *Selective outcome reporting*: Low  *Overall risk of bias*: Medium |
| McDonough 2002  *Location*:  United Kingdom  *Setting*:  Medical School  *EBP evaluated*: Exposure Therapy (ET)  *MH condition*: Phobias | *Training format:*  In-person lecture + in-person group tutorial (L+T): In-person lecture (time: 20 minutes) +in person, group tutorial (time: 90 minutes).  In-person lecture + online training (L+OLT): In-person lecture as described above + OLT (time: 90 minutes).  *Training content:*  L: Basic cognitive behavior therapy concepts and historical overview.  OLT: Participants given description of a case of agoraphobia and asked to 1) give the diagnosis, 2) define the problem in behavioral terms, 3) set treatment goals and ‘homework’ tasks, and 4) ‘troubleshoot’ problems. OLT took user the treatment rationale and case examples before helping to devise step-by-step personalized exposure program, complete with homework diaries. The OLT offered feedback. A ‘troubleshooting’ section identified reasons for case’s lack of progress in treatment and offered a choice of likely solutions.  T: Students worked through same 4 questions above in interactive way. | *N*=37  *Inclusion*: Third year medical students from King’s College Hospital Medical School London 5 weeks into their 6-week clinical attachment in psychiatry  *Exclusion*: No students were excluded  *Previous experience with EBP*: One-hour lecture on anxiety disorders but no previous instruction in graded exposure  *Discipline*:  Medical students = 100%  *Demographics*:  Mean age = NR  Female = 54.1%  Caucasian = NR | N/A – no clients in study | *Assessment measures*:  Knowledge: Measured by multiple choice questions consisting of 15 “stems” each containing 5 true or false statements for a total of 75 questions. The total score was percentage correct. Emphasis of questions was on testing student’s ability to apply principles of graded exposure to realistic clinical scenarios and recognize/avoid common pitfalls in treatment.  Satisfaction: Measured by asking participants two questions rated on 0 (not true at all) to 8 (very true) scale: (1) ‘I found the interactive teaching educational’ and (2) “I found the interactive teaching enjoyable”  *Assessment timeframe*:  Pre-training, post-training | *Random sequence generation*: Low; random number series used  *Allocation concealment*: Unclear  *Masking*: N/A – no maskeded assessments  *Incomplete outcome*  *data*: Low; no missing data  *Selective outcome reporting*: Low  *Overall risk of bias*: Medium |
| Miller 2004  *Location*:  United States  *Setting*:  Practice settings varied  *EBP evaluated*: Motivational Interviewing (MI)  *MH condition*:  Substance abuse | *Training format:*  In-person workshop (W): 2-day didactic and experiential workshop (time: 2 days)  Workshop + Feedback (W+F): W (described above) + emailed/mailed feedback on submitted audio tapes (time: NR)  Workshop + Coaching (W+C): W (described above) + up to 6, 30-minute individual coaching sessions by telephone in following 4 months (time: 3 hours)  W+F+C: All of the above components  Training manual + didactic videotapes (TM^+^): Received only manual and training tapes and asked to learn on their own for 6 months.  *Training content:*  *All groups received therapist manual and 7 training videotapes  W: expert-led workshop; no further training until after 8-month follow-up when given feedback on practice tapes  F: Feedback was sent to participants via email or hard copy, using a standard reporting form. The clinician feedback report provided summary measures from the MISC to provide trainees with specific information about their MI performance relative to expert models and to target levels of practice proficiency.  C: Coaching provided positive reinforcement, problem-solving consultation around difficulties encountered in the practice of MI, and demonstration and MI skills practice via role-played interviewing. Specific MISC performance feedback from practice samples was discussed only for participants as- signed to receive both feedback and coaching. | *N* = 140  *Inclusion*:  English-speaking US citizens or permanent residents; licensed health professionals in counseling, psychology, medicine, nursing, or social work; treating 5 or more clients with substance use disorders per week in individual counseling sessions; willing to travel to training site and submit 4 audiotapes of actual client sessions over one year period  *Exclusion*:  >8 hours prior training in MI  *Previous experience with EBP*: 48.2% had read MI book  *Discipline*:  NR  *Demographics*:  Mean age = 47.6 years  Female = 50.4%  Caucasian = 88.6% | Pre-workshop samples were audiotape of MI with actual clients being treated for a substance use disorder; Participants self-selected session to reflect their best performance. Clients consented but no client information was collected  Post-workshop audiotapes were of MI with a standard client actor portraying an offender on probation  Follow-ups audiotapes were of MI with actual clients | *Assessment measures:*  Competence: Measured by independent rating of session audiotapes (with real client pre-workshop and client actor post-workshop) on global MI spirit with the MI Skill Code (MISC); scores rated on 7-point Likert scale from 1 (low) to 7 (high)  Client outcomes: Measured by independent ratings of frequency of client responses of “change talk” and “resistance”  *Assessment timeframe*:  Pre-workshop; post-workshop; 4, 8, and 12 months follow-up.  *Note: only data from pre- and post-workshop and 4-month follow-up were extracted because additional training elements were subsequently added to the conditions, which may confound results.* | *Random sequence generation*: Low; urn randomization used  *Allocation concealment*: Low; participants and trainers unaware of condition assignment except for STC group  *Masking*: Unclear; notes that raters were unaware of timing of sample (pre-training or follow-up) but no information on if they were masked to condition assignment  *Incomplete outcome data*: High; 100% completed post-workshop assessment, 76%, 54%, and 45% completed 4-, 8-, and 12- month follow-up, respectively  *Selective outcome reporting*: Low  *Overall risk of bias*: Medium |
| Monson 2018  *Location*: Canada  *Setting*: Practice settings varied  *EBP evaluated*: Cognitive Processing Therapy (CPT)  *MH Condition*: Posttraumatic Stress Disorder | *Training Format:*  In-person workshop (W) (time: 2 days)  In-person workshop + consultation (W+C) (time: 2 days + 1-hour meetings, 1x per week for 6 months)  In-person workshop + consultation with session audio review (W+C^+^) (time: 2 days + -hour meetings, 1x per week for 6 months)  *Training Content:*  W: 2-day CPT workshop by CPT expert + CPT manual and related materials + access to resources available through the free CPT-web online training  C: Group consultation with CPT expert via web-based program over internet or phone. Discussion about provision of the CPT protocol, challenging cases, treatment obstacles, and specific issues raised by participants within each group.  C^+^: Consultation activities above +  one or two therapists per meeting presented segments of their audio recordings (typically 5–10 min) from a recent session and received feedback from other group members, as well as from the expert consultants. Therapists who did not present session content in a given week had the opportunity to provide a brief check-in about the progress of their current cases, and received input and feedback. | *N* = 81  *Inclusion*: Mental health therapists from Veterans Affairs Canada Operational Stress Injury Clinics, Canadian Forces mental health services, and the broader Canadian community who a) attended a standardized CPT workshop provided by Candice Monson, b) were licensed mental health therapists with psychotherapy in their scope of practice; c) were currently providing psychotherapy to individuals with PTSD; d) consented to be randomized; and e) were willing to provide audio recordings of therapy sessions, and measures of PTSD symptoms and psychosocial functioning from consenting clients.  *Exclusion*: NR  *Previous experience with EBP*: NR  *Discipline*:  PhD/PsyD: 41%  Master's: 41%  Bachelor's: 7%  MD: 5%  *Demographics*:  Mean age = 47.63 years  Female = 74%  Caucasian = NR | Clients had to a) be diagnosed with PTSD by their therapist according to DSM-IV criteria, b) and have a score of ≥ 50 on the PCL-IV, and c) consent to participate in CPT, have their sessions audio recorded and reviewed by independent fidelity raters, as well as potentially other therapists during consultation.  Clients were ineligible if they had current uncontrolled psychosis or bipolar disorder, substance dependence (abuse was permitted), imminent suicidality or homicidality, or significant cognitive impairment.  *Demographics*:  Mean age = 39.4 years  Female = 52.0%  Caucasian = 88.0% | *Assessment measures:*  Client outcomes: Measured by PCL-IV, a 17-item self-report measure of PTSD symptoms. Total score ranges from 0 to 85 with cut-score of 50 and above indicating likely PTSD. | *Random sequence generation*: Unclear  *Allocation concealment*: Unclear  *Masking*: Unclear if adherence raters masked to condition  *Incomplete outcome data*: Low; 60% of those randomized participated. No Participants dropped and 1 participant did not supply client outcomes.  *Selective outcome reporting*: Low  *Overall risk of bias*: Medium |
| Moyers 2008  *Location*:  United States  *Setting*:  United States Air Force  *EBP evaluated*: Motivational Interviewing (MI)  *MH condition*:  Substance abuse | *Training format:*  Workshop (W): 2-day, in-person  Workshop + Feedback + Consultation (W+F+C): Workshop + feedback on post-training work sample + up to 6 consultation phone calls (30 minutes, approx. 2 weeks apart)  Training manual + didactic videotapes (TM^+^): Self-study of manual and videotapes (time: NR)  *Training content:*  W: Used “learning-to-learn” model, which introduced MI concepts, methods for improving MI practice based on client responses.  F+C: Written feedback on post-training work sample + up to 6 consultation phone calls with role-plays and specific topics to enhance MI skills | *N* =129  *Inclusion*:  Behavioral health Participants (included degree in psychology, medicine, social work, nursing, counseling, or certification in alcohol and drug or substance abuse treatment) working directly with substance abuse clients in the US Air Force.  *Exclusion*: NR  *Previous Experience with EBP*: NR  *Discipline*: NR  *Demographics*:  Mean age = 36.3 years  Female = 51.3%*  Caucasian = 55.7%  *** significantly higher percentage of females in TM^+^ condition. | Pre-training and follow-up assessments of MI used audiotape of actual client sessions chosen by participants. Client characteristics NR  Post-workshop assessment of MI was conducted with standardized client actors | *Assessment measures*:  Adherence: Measured by number of MI adherent behaviors by single, masked rater using MITI  Competence: Measured by Global MI spirit also rated on the MITI. Clinical proficiency for MI spirit is defined as rating of 5 or above.  *Assessment timeframe*:  Pre-training, post-training, 4-, 8-, and 12-month follow ups  *Participants in the TM^+^ condition submitted audiotapes of client sessions before they completed the workshop. | *Random sequence generation*: Unclear  *Allocation concealment*: Unclear  *Masking*: Low; Raters were masked to training condition  *Incomplete outcome data*: High; 90% submitted post-training tape; 68%, 58%, and 38% completed 4-, 8-, and 12-month follow-up, respectively  *Selective outcome reporting*: Low  *Risk of bias*: Medium |
| Puspitasari 2017  *Location*:  United States  *Setting*:  Graduate school  *EBP evaluated*: Behavioral Activation (BA)  *MH condition*: Depression | *Training format:*  Online training (OLT): Self-paced, four 40-minute presentations with audio guide. Presentations delivered weekly to participants (time: 2.67 hours)  Distance workshop (DW): Four, 90-minute trainer-led interactive presentations (time: 6 hours)  *Training content:*  OLT: BA with audio guide from trainer. Presentations delivered weekly to participants and they were encouraged to practice BA skills, use in their own lives and with clients.  DW: 90-minute interactive presentations in which expert interacted with 8-10 participants via training website. Participants role-played BA skill, received feedback from expert and other trainees, and given between-session assignment to integrate BA skills in own lives and with clients. | *N* = 77  *Inclusion*:  Undergoing supervision with a licensed individual; current graduate-level student in: clinical psychology, counseling psychology, social work, nursing, or psychiatry residency; actively seeing depressed outpatient clients as part of training; able to comply with study procedures (i.e., computer with high speed internet access)  *Exclusion*: NR  *Previous experience with EBP*:  28% of participants reported using BA strategies pre-training  *Discipline*:  Clinical psychology student = 54.5%  Counseling psychology student = 14.3%  Social work student = 11.7%  Psychiatry resident = 19.5%  *Demographics*:  Mean age = 30.3 years  Female = 76.6%  Caucasian = 71.4% | Two simulated clients played by trained assessor portraying clients with depression. | *Assessment measures:*  Competence: Measured by independent coders using audiotapes of role-play with simulated client on the 28-item BA Skills Assessment (BASA) scale. Each item was rated on 6-point Likert Scale from 0 (not at all competent) to 6 (extremely competent). Scores were totaled with a range of scores from 0-168.  Adoption: Measured by participant’s self-report of whether or not they implemented any of the core BA strategies with at least one client in the preceding week.  Satisfaction: Measured by satisfaction with each training session using the Training Satisfaction Questionnaire (TSQ) consisting of 6 questions rated from 1 (poor) to 5 (excellent). Total scores ranged from 6-30.  Assessment Timeframe: Pre-training, post-training, 3-month follow-up  *Assessment timeframe:*  Pre-training, post-training, 6-week follow-up | *Random sequence generation*: Low; stratified randomization used  *Allocation concealment*: Unclear  *Masking*: Low; raters masked to participants’ condition  *Incomplete outcome data*: High; 53% of participants completed follow-up  *Selective outcome reporting*: Low  *Overall risk of bias*: Medium |
| Rakovshik 2016  *Location*:  Russia and Ukraine  *Setting*:  Varied  *EBP evaluated*: Cognitive Behavioral Therapy (CBT)  *MH condition*:  General with modules on panic disorder with agoraphobia (PD) and obsessive-compulsive disorder (OCD) | *Training format:*  OLT + consultation worksheet (OLT+CW): Self-paced OLT (time: 20 hours over 3 months). CW time NR.  Online training + consultation worksheet + remote supervision (OLT+CW+RS): OLT described above + three, 30-minute individual supervision sessions monthly (time: 90 minutes). CW time NR.  Delayed-training control (NT): Participants did not receive the training until all data collection was complete.  *Training content:*  OLT: Included three modules: (1) CBT theory formulation and assessment, (2) general CBT skills and (3) protocols for treating PD and OCD. Presentations included videos of instruction and clinical role plays, with simultaneous PowerPoint presentation delivered by expert.  CW: Translated version of Christine Padesky’s worksheet which asks troubleshooting questions about case conceptualization and treatment progress. Completed monthly during training and submitted to research team. If the worksheet was not returned, then one additional e-mail was sent as a reminder.  RS: Individual skype supervision sessions monthly. Supervision focused on developing and updating a CBT conceptualization and review of treatment plan. | *N* = 61  *Inclusion*:  Practicing mental health Participants from Russia and Ukraine, otherwise NR  *Exclusion*: NR  *Previous Experience with EBP*: Average of 87.1 (SD = 218.2) hours of prior CBT study. Stratified based on previous CBT training (cut point 38 hours)  *Discipline*:  Psychologist = 80%  Psychiatrist = 8%  Psychiatrist-psychotherapists = 12%  *Demographics*:  Mean age = NR  Female = 70%  Caucasian = NR | Clients in routine clinical practice, characteristics NR | *Assessment measures:*  Competence: Measured by ratings of recorded therapeutic sessions by study authors via the 11-item Cognitive Therapy Scale (CTS), with each skill rated on a 6-point Likert scale from 0 (poor) to 6 (excellent). A mean per item was calculated as the reported measure of competence.    Assessment timeframe:  Pre-training, Mid-training, Post-training | *Random sequence generation*: Low; random permutated block method used  *Allocation concealment*: Low; external online service informed participants and investigator of allocation simultaneously via email  *Masking*: High; not all raters masked to participants’ condition  *Incomplete outcome data*: High; 77% completed post-training follow-up  *Selective outcome reporting*: Low    *Overall risk of bias*: High |
| Rawson 2013  *Location*: South Africa  *Setting*:  Addiction treatment centers  *EBP evaluated*: Cognitive Behavioral Therapy (CBT)  *MH Condition*: Substance abuse | *Training format:*  In-person Workshop + in-person coaching (W+C): 3-day training + six bi-weekly, 90-minute in person expert coaching sessions (ended at 12-week post-training)  Distance Workshop + telephone coaching (DW+C): live workshop through televised, interactive instructional platform + coaching sessions over telephone conference call (ended at 12-week post-training)  Training manual + orientation (TM^+^): manual + 2-hour orientation  *Training content:*  W+C: 3-day workshop included didactic training and role-play of CBT methods. In-person expert coaching sessions occurred in participants’ clinics.  DW+C: Same workshop described above but viewed over televised, interactive platform. Coaching was also the same but conducted over telephone.  TM^+^: Manual provided + 2-hour orientation to manual and guidelines for its use | *N =* 143  Randomization at clinic level.  *Inclusion*:  Voluntary participation by centers director or staff with ≥3 clinician volunteers; practicing addition clinicians willing to be randomly assigned, participate in training sessions, and have their sessions audiotaped  *Exclusion*: NR  *Previous experience with EBP*: NR  *Discipline*:  Social worker=49%  Other: NR  *Demographics*:  Mean age = 38.1 years  Female = 75%  Caucasian = 36.4% | NR – No identifying information collected from clients | *Assessment measures:*  Adherence: Measured by masked, independent raters listening to session audiotapes of actual therapy sessions and rated adherence on the 55-item Yale Adherence and Competence Scale (YACS). Each item was rated on a 7-point Likert scale from 1 (not at all) to 7 (high frequency). Scores averaged for range from 1 to 7.  Competence: Measured by the YACS (described above) with each item rated on a 7-point Likert scale from 1 (very poor) to 7 (excellent). Scores averaged for range from 1 to 7.  Cost: Measured by delivery-specific expenses for each training condition, including materials, travel costs, expert trainer visits, personnel costs, and costs of videoconferencing service. Total costs were tabulated for all participants within each training condition  Knowledge: Measured by 28-item multiple choice questionnaire. Scores were totaled for a range from 0-28)  *Assessment timeframe*:  Pre-training, 4-, 8, 12, and 24-weeks post-training | *Random sequence generation*: Low; urn randomization  *Allocation concealment*: Unclear  *Masking*: Low, raters masked to participants’ condition  *Incomplete outcome data*: High, 62.9% completed all follow-up assessments  *Selective outcome reporting*: Low  *Overall risk of bias*: Medium |
| Ruzek 2014  *Location*:  United States  *Setting*:  Veterans Health Administration (VHA)  *EBP evaluated*:  Cognitive Behavioral Therapy (CBT) skills (motivational enhancement, goal setting, behavioral task assignment)  *MH condition*: Posttraumatic Stress Disorder | *Training format:*  Online training (OLT): OLT (time: 6 hours)  OLT + telephone consultation (OLT+TC): OLT described above + up to six, 45-60-minute small group sessions (time: maximum 6 hours).  No training control (NT): Participants free to participate in any training activities they would they would otherwise receive, including local continuing education activities, conferences, or other formal training programs.  *Training content:*  OLT: Three modular courses focused on a) motivational enhancement, b) goal setting, and c) behavioral task assignment. Web modules included interactive exercises, audio-streamed skills demonstrations, case examples, downloadable materials, and bibliographies.  TC: Manualized, small group consultation sessions. Clinicians were asked to apply the skills with their PTSD cases, and with other clients as deemed appropriate. | *N* = 168  *Inclusion*:  Full-time VHA mental health clinicians with direct care responsibilities for veterans with PTSD in a clinic providing PTSD treatment.  *Exclusion*: NR  *Previous* *Experience with EBP*:  NR. (Participants were stratified by self-rated degree of expertise in CBT)  *Discipline*:  Master’s level = 57.1%  Doctoral-level degree = 36.2  *Demographics*:  Mean Age = 48.8 years  Female = 69.6%  Caucasian = 73.8% | Simulated clients (clinical psychology graduate students working in VHA who portrayed an individual with PTSD) | *Assessment measures:*  Adherence: Measured by rating based on role-play with standardized client and rated by masked graduate student research staff. Skill adherence was measured on 3-point scale (0 = no adherence, 1 = partial adherence, 2 = full adherence) on each of the three skills (ME, BTA, GS) were also calculated.  Adoption: Measured by participant’s self-report of how often they used 12 specific subskills in clinical management of PTSD clients during the past 30 days on a 5-point Likert scale from 1 = Almost Never to 5 = Very often. Overall Adoption was mean of 12-items.  Knowledge: Measured by 12-item multiple choice test. Total knowledge score comprised of number of correct responses. Subscales for each skill (ME, BTA, GS) were also calculated.  *Assessment timeframe*:  Pre-training, post-training | *Random sequence generation*: Low; Automated randomization system  *Allocation concealment*: Unclear  *Masking*: Low; Simulated clients and raters masked to participants’ condition  *Incomplete outcome data*: Medium; 83% completed post-training assessment  *Selective outcome reporting*: Low  *Overall risk of bias*: Medium |
| Smith 2012  *Location*:  United States  *Setting*:  Substance use community treatment programs  *EBP evaluated*: Motivational Interviewing (MI)  *MH condition*:  Substance abuse | *Training format:*  In-person workshop (W): 2-day workshop  In-person workshop + session audio review (W+F): workshop + supervision via in one-hour telephone call (time: 5 hours).  In-person workshop + live teleconferencing supervision (W+LS): Workshop + real-time supervision via telephone ear-piece (time: 5 hours).  *Training content:*  W: 2-day MI training workshop, which included didactic and interactive learning exercises (role-plays)  F: 5 recorded practice counseling sessions with simulated clients over 7 weeks. Tape reviewed by supervisor and given feedback in one-hour supervision call.  LS: 5 practice counseling sessions with simulated client over 7 weeks. Supervisor provided real-time feedback via ear-piece. | *N* = 97  *Inclusion*:  Between 18 and 75 years old, provide counseling services directly to clients, be employed at least half time at their substance abuse treatment facility prior to enrollment in study.  *Exclusion*:  Attended MI workshop in past 3 months, received training to be an MI trainer, participated in previous MI research trial.  *Previous experience with EBP*: NR  (Years in substance abuse field: 8.2)  *Discipline*: NR.  High school = 15.0%  College = 43.4%  Graduate degree = 39.3%  *Demographics*:  Mean age = 43.9 years  Female = 65.0%  Caucasian = 28.6% | Simulated client for post-workshop practice supervision sessions (actor portraying clients at various stages of change).  Treatment-enrolled clients for outcome analyses:  Inclusion: Between 18 and 70 years old, new to treatment program or currently enrolled but new to counselor doing interview, using alcohol or other illicit substances other than nicotine at least once per week in 30 days prior, able to speak and understand English, willing to have session taped.  Exclusion: In need of immediate treatment for severe psychiatric disorder/medical condition or reported legal problems that could result in incarceration for >4 weeks. | *Assessment measures:*  Competence: Measured by global spirit and empathy rating from 1 (low) to 7 (high) via the MITI from 20-minute segment of a counseling session with treatment-enrolled client. Proficiency: beginner level = 5; competent = 6 or 7  Adherence: Measured by behavior count rated on MITI rated form 20-minute segment of a counseling session with treatment-enrolled client. Percent MI adherent is total # of MI adherent behaviors divided by total number of behaviors (adherent + non-adherent). Proficiency = 100%    *Assessment timeframe*:  Pre-workshop, post-workshop,  post-supervised training (or control) (week 8), follow-up (week 20) | *Random sequence generation*: Low; Computer generated randomly permuted blocks.  *Allocation concealment*: Unclear; randomization assignments distributed by counselors at end of workshop  *Masking*: Low; raters were masked to participants’ condition  *Incomplete outcome data*: Low; 91% had data at one or both outcome assessments.  *Selective outcome reporting*: Low  *Overall risk of bias*: Low |
| Weingardt 2006  *Location:*  United States  *Setting*:  Participant practice settings varied  *EBP evaluated:* Cognitive Behavioral Therapy (CBT)  *MH condition*: Substance abuse | *Training format:*  Online training (OLT): Self-directed OLT in a designated computer lab. (time: one 60-minute session)  In-person workshop (W): (Time: one 60-minute session)  Delayed training control (NT): Video tape (time: one 60-minute session)  *Training content:*  OLT: designed to train practitioners in the “Coping with Craving” module from the National Institute on Drug Addiction treatment manual “A Cognitive-Behavioral Approach: Treating Cocaine Addiction”  W: Expert presented content directly from “Coping with Craving” module exactly as written  NT: Participants watched an unrelated videotape. | *N* = 166  *Inclusion*:  Practicing substance abuse counselor evidenced by a valid state license/credential or a recent check stub  *Exclusion*: NR  *Previous experience with EBP*: 82.5% of participants familiar with CBT  *Discipline*:  Substance abuse counselors=100%  *Demographics*:  Mean age = 44.4 years  Female = 54.8%  Caucasian = 54.8% | N/A – no clients in study | *Assessment measures:*  Knowledge: Measured by 17-item multiple choice test that covered three domains of the “Coping with Craving” module (understanding/describing craving, identifying triggers/avoiding cues, strategies for coping with craving).  *Assessment Timeframe*: Pre-training and post-training | *Random sequence generation*: Unclear  *Allocation concealment*: Low; participants given numbered and colored folders from central registration desk  *Masking*: N/A – no masked assessments  *Incomplete outcome data*: Unclear; attrition NR  *Selective outcome reporting*: Low  *Overall risk of bias*: Medium |
| Zatzick 2014  *Location*:  United States  *Setting*:  Level I trauma centers  *EBP evaluated*: Motivational Interviewing (MI)  *MH condition*: Problematic alcohol use  Note: Some information from study reported in Darnell 2016 | *Training format:*  In-person workshop + feedback + coaching (W+F+C): 1-day, in-person workshop followed by four, 30-minute telephone coaching sessions + written feedback via e-mail over 6 months post-workshop (time: 1 day + 2 hours coaching)  No Training (NT): Participants at were permitted to implement mandated alcohol screening and brief intervention with assistance of any available guidelines, training materials, other resources  *Training content:*  W+F+C: The in-person workshop emphasized MI skill development and the importance of spending adequate time conducting interventions by the bedside with injured inpatients. The following trainer-led telephone coaching sessions included MI skills practice plus written feedback via e-mail over 6 months post-workshop. | *N* = 40  Randomization at site (N=20) level.  *Inclusion*:  American College of Surgeons-verified level I trauma centers; “middle adopter” sites (i.e., demonstrated interest in alcohol service development but without well-established SBI services)  *Exclusion*:  Sites classified as “innovators/early adopters” or “laggard/late adopters”  *Previous Experience with EBP*: NR  *Discipline*:  Nursing = 47.5%*  Social Work = 37.5%*  Other = 15.0%  *Workshop condition had significantly fewer nurses and more social workers than no training condition.  *Demographics*:  Mean age = NR  Female = 95.0%  Caucasian = 90.0% | N=878  Inclusion: Male or female survivors of intentional and unintentional injuries; age ≥18; positive blood alcohol concentration test upon admission;  score of 15/15 on Glasgow Coma Scale; score ≥7 on mental status examination  Exclusion: Self-inflicted intentional injuries that constituted suicide attempts; non-English speaking; prisoners; AUDIT score ≥20  Demographics:  Mean age = 36.9 years  Female = 23.7%  Caucasian = 62.1%  TBI: 50.3%  Mean days in hospital: 6.1 | *Assessment measures:*  Adherence: Measured by 7, 20-minute standardized client-actor telephone interviews that increased in clinical difficulty. % MI adherence rated on MITI; percent MI adherent is total # of MI adherent behaviors during client encounter divided by total number of behaviors (adherent + non-adherent)  Competence: Measured by Global MI spirit from MITI measured with rating on 5 point Likert scale, with higher score indicating more MI-consistent  *Assessment Timeframe*:  Pre-training, post-training, 1-, 4-, 7-, 17-, and 27-month follow ups  Client outcomes: Percentage met criteria for hazardous drinking as measured by the Alcohol Use Disorders Identification Test (AUDIT) score (≥8 for men and ≥5 for women)  *Assessment Timeframe*:  Hospital admission, 6 months, and 12 months | *Random sequence generation*: Low; block randomization using random number generator by statistician  *Allocation concealment*: Low; site randomization conducted by masked research coordinator  *Masking*: Low; standardized clients and outcome assessors were masked to condition  *Incomplete outcome data*: High; 28.5% of clients were not assessed at 12 months.  *Selective outcome reporting*: Low  *Overall risk of bias*: Medium |

**Table 2. Overview of Included Studies’ Findings (*k*=28)**

| **Study (first author and year)**  **Provider *N***  **Risk of Bias** | **Training methods evaluated** | **EBP**  **Mental health condition** | **Provider satisfaction w/ training** | **Provider treatment knowledge** | **Provider adherence/ skill acquisition** | **Provider competence** | **Provider fidelity** | **Provider EBP adoption** | **Client clinical outcomes** | **Costs of training** |
| --- | --- | --- | --- | --- | --- | --- | --- | --- | --- | --- |
| Bearman 2017  *N*=40  ROB=Med. | In-person workshop + supervision as usual (W+SAU)  In-person workshop + experiential supervision (W+SUP^+^) | Cognitive Behavioral Therapy  Youth depression |  |  |  | X^a^  W+SUP^+^ > W+SAU  *d* = .64 | X^a^  W+SUP^+^ > W+SAU  *d* = .63 and .70 |  |  |  |
| Beidas 2012  *N*=115  ROB=Low | In-person didactic workshop + virtual consultation (W+VC)  In-person experiential workshop + virtual consultation (W^+^+VC)  Online training + virtual consultation (OLT+VC) | Cognitive Behavioral Therapy, Coping Cat protocol  Youth anxiety | X^c^  W^+^ & W > OLT  *p* < .05 and *p* < .001 | X^b^  nsd conds.  All ↑ pre-post train.  *p* < .001 | X^a^  nsd conds.  All ↑ pre-post train.  *p* < .001 | X^a^  nsd conds.  All ↑ pre-post train.  *p* < .001 |  |  |  |  |
| Bennett-Levy 2012  *N*=49  ROB=Med. | Online training (OLT)  Online training + supportive calls (OLT+SC) | Cognitive Behavioral Therapy  Depression, panic disorder w/ agoraphobia, generalized anxiety disorder |  | X^b^  nsd conds.  All ↑ pre-post train.  *p* < .001 | X^c^  nsd conds.  All ↑ pre-post train.  *p* < .001 |  |  | X^c^  nsd conds.  All ↑ pre-post train.  *p* < .001 |  | X |
| Chu 2017  *N*=35  ROB=Med. | Online training + online expert streaming consultation (OLT+ES)  Online training + in-person peer consultation (OLT+PC)  Online training + fact sheet self-study (OLT+FS) | Cognitive Behavioral Therapy  Youth anxiety | X^c^  nsd conds. | X^b^  nsd conds.  All ↓ pre-post consult.  *d* = -1.42 |  |  |  | X^c^  nsd conds.  All ↑ pre-post consult.  *d* = .8 |  |  |
| Cohen 2016  *N*=81  ROB=Med. | Online training + online consultation course (OLT+OC)  Online training + in person workshop + phone consultation (OLT+W+PhC) | Trauma- Focused Cognitive Behavioral Therapy  PTSD and depression |  |  | X^c**^  OLT+W+C > OLT+OC  *p* < .001 |  |  |  | X^e^  OLT+W+C > OLT+OC  *p* < .05 |  |
| Cooper 2017  *N*=156  ROB=Med. | Online training (OLT)  Online training + supportive calls (OLT+SC) | Cognitive Behavioral Therapy  Eating disorders |  | X^b*^  nsd conds.  All ↑ pre-post train.  *p* < .001 |  |  |  |  |  |  |
| Dimeff 2009  *N*=150  ROB=Med. | Online training (OLT)  In-person workshop (W)  Treatment manual (TM) | Dialectical Behavior Therapy  Borderline personality disorder | X^c^  OLT & W > TM, *d*=.73 – 1.6  OLT & W nsd | X^b^  OLT > W & TM, *d*=.37 – .52  W & TM nsd  All ↑ pre-post train.  *p* < .001 | X^a^  nsd conds.  All ↑ pre-post, follow-up, *p* < .001 | X^a^  nsd conds.  All ↑ pre-post, follow-up  *p* < .001 |  | X^a^  nsd conds.  Assessed follow-up only |  |  |
| Dimeff 2011  *N*=132  ROB=Low | Online training (OLT)  Placebo online training (pOLT)  Treatment manual (TM) | Dialectical Behavior Therapy  Borderline personality disorder | X^c^  Acceptability:  OLT & TM > pOLT  *d*= .56 – 1.3  OLT > TM  *d*= .65 – .80  Usability:  OLT & pOLT> MT  *d*= .37 – .75 | X^b^  OLT and TM > pOLT  *d*= 1.5 – 3.5  OLT > TM follow-up only, *d*=.36    All ↑ pre-post, follow-up, *p* < .001 |  |  |  | X^c^  OLT > pOLT all timepoints  *p* < .05  TM > pOLT 2-week follow-up  *p* < .05  OLT > TM at 7-week follow-up  *p* < .05  Assessed follow-up only |  |  |
| Dimeff 2015  *N*=172  ROB=Med. | Online training (OLT)  In-person workshop (W)  Treatment manual (TM) | Dialectical Behavior Therapy  Borderline personality disorder | X^c^  W > OLT & TM, *p* < .001 | X^b^  OLT > W & TM, *d*= .46 – .62  All ↑ pre-post, follow-up, *p* < .01 |  | X^a^  nsd conds.  All ↑ pre-post, follow-up, *p* < .001 |  | X^c^  nsd conds.  All ↑ pre-post, follow-up, *p* < .05 |  |  |
| Fu 2015  *N*=34  ROB=Low | In-person workshop (W)  In-person workshop + booster sessions + peer coaching (W+BS+C) | Motivational Interviewing  Smoking cessation |  | X^b^  W+BS+C > W, *p* < .05 | X^a^  W+BS+C > W, *p* < .01 | X^a^  W+BS+C > W, *p* < .01 |  |  |  |  |
| Gega 2007  *N*=92  ROB=Low | Online training (OLT)  In-person workshop (W) | Exposure therapy  Phobias | X^c^  nsd conds. | X^b^  nsd conds.  All ↑ pre-post train.  *p* < .001 |  |  |  |  |  |  |
| Harned 2011  *N*=46  ROB=Med. | Online training (OLT)  Online training + motivational interviewing (OLT^+^)  Placebo online training (pOLT) | Exposure therapy  Anxiety disorders | X^c^  Acceptability:  OLT & OLT^+^ > pOLT  *p* < .05  OLT & OLT^+^  nsd  Usability:  nsd conds. | X^b^  OLT & OLT^+^ > pOLT  *d*= 1.7 – 3.3  OLT & OLT^+^ nsd  All ↑ pre-post-train., follow-up  *p* < .001 |  |  |  | X^c^  nsd conds.  No sig. change pre-post train. |  |  |
| Harned 2014  *N*=181  ROB=Med. | Online training (OLT)  Online training + motivational enhancement (OLT^+^)  Online training + motivational enhancement + learning community (OLT^+^+LC) | Exposure therapy  Anxiety disorders | X^c^  Motivational enhancement:  OLT^+^+LC > OLT^+^, *p* < .05  nsd in OLT component rating | X^b^  OLT^+^+LC > OLT & OLT^+^  *d*=.32 – .39  OLT & OLT^+^ nsd  All ↑ pre-post, follow-up, *p* < .001 |  | X^a^  OLT^+^+LC > OLT  *d*=.39  All ↑pre-post, follow-up, *p* < .001 |  | X^c^  nsd conds.  All ↑ pre-post, follow-up, *p* < .001 |  |  |
| Henggeler 2008  *N*=30  ROB=Med. | Workshop (W)  Workshop + Intensive Quality Assurance (W+IQA) | Contingency Management  Marijuana abuse |  |  | X^d^  CBT techniques:  W+IQA > W  *p* < .05  CM Monitoring:  nsd conds.  No sig. change pre-post train. |  |  |  |  |  |
| Hubley 2015  *N*=49  ROB=Low | Online training (OLT)  Online placebo control training (pOLT) | Behavioral Activation  Depression |  | X^b^  OLT > pOLT  *d* = 1.16  All ↑ pre-post, follow-up  *p* < .001 |  |  |  |  |  |  |
| Larson 2013  *N*=127  ROB=Med. | Online training + supervision (OLT+S)  Training manual + supervision (TM+S) | Cognitive Behavioral Therapy  Substance abuse |  |  | X^a^  nsd conds.  Only TM+S ↑ pre-post  *p* < .05 |  |  |  |  |  |
| Martino 2011  *N*=92  ROB=Med. | In person workshop by expert + consultation (EW+C)  In person workshop by trainee + consultation (TW+C)  Treatment manual + didactic materials (TM^+^) | Motivational Interviewing  Substance abuse |  |  | X^a^  Client:  nsd conds.  Role-play:  EW+C & TW+C > TM^+^  *d* = .63-1.2  EW+C & TW+C nsd | X^a^  Client:  EW+C > TM^+^  *d* = 1.05-1.19  TW+C & TM^+^ nsd  Role-play:  TW+C > TM^+^  *d* = .54-.72  EW+C > TW+C, *p* < .01 |  |  |  |  |
| McDonough 2002  *N*=37  ROB=Med. | In-person lecture + online training (L+OLT)  In-person lecture + in-person group tutorial (L+T) | Exposure therapy  Phobias | X^c^  Educational:  L+T > L+OT  *p* < .05  Enjoyable:  L+T > L+OT  *p* < .001 | X^b^  nsd conds.  All ↑ pre-post  *p* < .001 |  |  |  |  |  |  |
| Miller 2004  *N*=140  ROB=Med. | In-person workshop (W)  In-person workshop + audio review and feedback (W+F)  In-person workshop + coaching (W+C)  In-person workshop + audio review and feedback + coaching (W+F+C)    Training manual + didactic videotapes (TM^+^) | Motivational Interviewing  Substance abuse |  |  | X^a^  W, W+F, W+C, W+F+C > TM^+^  *p* < .001  W, W+F, W+C, W+F+C nsd  W, W+F, W+C, W+F+C ↑ pre- to 4-month follow-up, *p* < .001-.05 | X^a^  W, W+F, W+C, W+F+C > TM^+^  *p* < .01  W+F, W+C, W+F+C > W  *p* <.05  W, W+F, W+C, W+F+C ↑ pre- to 4-month follow-up, *p* < .001-.05 |  |  |  |  |
| Monson 2018  *N*=81  ROB=Med. | In-person workshop (W)  In-person workshop + consultation (W+C)  In-person workshop + consultation with session audio review (W+C^+^) | Cognitive Processing Therapy  Posttraumatic stress disorder |  |  |  |  |  |  | X^e^  W+C > W  *d*=0.83 |  |
| Moyers 2008  *N*=129  ROB=Med. | In-person workshop (W)  In-person workshop + feedback + consultation (W+F+C)  Training manual + didactic videotapes (TM^+^) | Motivational Interviewing  Substance abuse |  |  | X^a^  nsd conds.  All ↑ pre-post train., *d* =.53-1.76 | X^a^  W+F+C and W > TM^+^, *p* <.001  W+F+C & W nsd  All ↑ pre-post train., *d* =1.7-2.5 |  |  |  |  |
| Puspitasari 2017  *N*=77  ROB=Med. | Distance workshop (DW)  Online training (OLT) | Behavioral Activation  Depression | X^c^  nsd conds. |  |  | X^a^  DW > OLT  *p* <.05  All ↑ pre-post, follow-up, *p* <.05 |  | X^c^  nsd conds.  All ↑ pre-post, follow-up, *OR = 5.1-5.8* |  |  |
| Rakovshik 2016  *N*=61  ROB=High | Online training + consultation worksheet (OLT+CW)  Online training + consultation worksheet + remote supervision (OLT+CW+S)  Delayed training control (NT) | Cognitive Behavioral Therapy  Panic disorder w/ agoraphobia, obsessive-compulsive disorder |  |  |  | X^a^  OLT+CW+S > OLT+CW & NT  *d* =.97 & 1.04 |  |  |  |  |
| Rawson 2013  *N*=143  ROB=Med. | In-person workshop + in-person coaching (W+C)  Distance workshop + telephone coaching (DW+TC)    Training manual + orientation (TM^+^) | Cognitive Behavioral Therapy  Stimulant dependence |  | X^b^  W+C > TM^+^  *p*<.05  W+C & DW+TC nsd  DW+TC & TM^+^ nsd | X^a^  W+C & DW+TC > TM^+^  *p* <.001  W+C & DW+TC nsd | X^a^  W+C & DW+TC > TM^+^  *p* <.01  W+C > DW+TC  *p* <.01  All ↑ pre-post  *p* <.001 |  |  |  | X |
| Ruzek 2014  *N*=168  ROB=Med. | Online training (OLT)  Online training + telephone consultation (OLT+C)  No training control (NT) | Cognitive Behavioral Therapy skills  Posttraumatic stress disorder |  | X^b^  OLT+C & OLT > NT on 2/3 skills  *p* <.001  OLT+C > OLT on 1/3 skills | X^a^  OLT+C & OLT > NT on 2/3 skills  *p* <.01  OLT+C > OLT on 1/3 skills, *p* <.001 |  |  | X^c^  nsd conds.  All ↑ pre-post  sig. NR |  |  |
| Smith 2012  *N*=97  ROB=Low | In-person workshop (W)  In-person workshop + session audio review (W+AR)  In-person workshop + live teleconferencing supervision (W+LS) | Motivational Interviewing  Substance abuse |  |  | X^a^  W+LS > W  *d*=.59  W+LS & W+AR nsd | X^a^  Spirit  W+LS & W+AR> W  *d*=1.01 & .53  Empathy  W+LS > W  *d*=.74 |  |  |  |  |
| Weingardt 2006  *N*=166  ROB=Med. | In-person workshop (W)  Online training (OLT)  Delayed training control (NT) | Cognitive Behavioral Therapy  Cocaine addiction |  | X^b^  W & OLT > NT, *p* < .01  W & OLT nsd  Only W and OLT ↑ pre-post, *p* < .05 |  |  |  |  |  |  |
| Zatzick 2014  *N*=40  ROB=Med. | In-person workshop + feedback + coaching (W+F+C)  No training (NT) | Motivational Interviewing  Problem drinking |  |  | X^a^  W+F+C > NT, *p* < .05 | X^a^  W+F+C > NT, *p* < .001 |  |  | X^e^  W+F+C > NT, *p* < .05 | X |

*Note.* ^a^ = trained observer rating; ^b^ = written test; ^c^ = self-report, ^d^ = client rating of therapist, ^e^ = client self-report. * = study reported as clinician competence; however, met our definition of treatment knowledge. ** = study reported as clinician fidelity; however, met our definition of treatment adherence. nsd = no significant difference. Effect sizes reported when available.

**Table 3a. Study Outcomes: Participant Adherence/EBP Skill Acquisition, Competence, and Fidelity**

| **Study**  **EBP and MH condition**  **Assessment Timeframe** | **Participant adherence/**  **EBP skill acquisition**  ***Mean (SD)***** | | | | | | | | | | | | | | | **Participant competence**  ***Mean (SD)***** | | | | | | | | | | | | **Participant fidelity**  ***Mean (SD)***** | |
| --- | --- | --- | --- | --- | --- | --- | --- | --- | --- | --- | --- | --- | --- | --- | --- | --- | --- | --- | --- | --- | --- | --- | --- | --- | --- | --- | --- | --- | --- |
| Bearman 2017 | NR | | | | | | | | | | | | | | | ***CBT Expertise (range 1-3)*** | | | | | | | | | | | | ***TIEBI (range 0-4)*** | |
|  |  |  |  |  |  |  |  |  |  |  |  |  |  |  |  | **W+SAU**  Pre: 1.2 (0.4)  Post: NR  Follow-up: NR | | | | | **W+SUP^+^**  Pre: 1.1 (0.4)  Post: NR  Follow-up: NR | | | | | | | **W+SAU**  Pre: 1.3 (0.7)  Post: NR  Follow-up: NR | **W+SUP^+^**  Pre: 1.2 (0.6)  Post: NR  Follow-up: NR |
|  |  |  |  |  |  |  |  |  |  |  |  |  |  |  |  | Both conditions had significant improvements in CBT expertise pre- to post-training and no significant difference between conditions was found. From post-training through supervision follow-up, the W+ SUP^+^ group had significantly greater increases in CBT expertise. Participants were rated at “novice” level post-training. | | | | | | | | | | | | Both conditions had significant improvements in cognitive restructuring fidelity pre- to post-training and no significant difference between conditions was found. From post-training through supervision follow-up, the W+ SUP^+^ group had significantly greater increases in fidelity. Participants were rated as having “adequate but not optimal” cognitive restructuring fidelity. | |
|  |  |  |  |  |  |  |  |  |  |  |  |  |  |  |  | ***CBT Global Competence (range 1-10)*** | | | | | | | | | | | |  |  |
|  |  |  |  |  |  |  |  |  |  |  |  |  |  |  |  | **W+SAU**  Pre: 2.7 (1.3)  Post: NR  Follow-up: NR | | | | | **W+SUP^+^**  Pre: 2.7 (1.2)  Post: NR  Follow-up: NR | | | | | | |  |  |
|  |  |  |  |  |  |  |  |  |  |  |  |  |  |  |  | Both conditions had significant improvements in Global CBT competence pre- to post-training and no significant difference between conditions was found. From post-training through supervision follow-up, the W+ SUP^+^ group had significantly greater increases in global CBT competence. Participants were rated at “novice” level post-training. | | | | | | | | | | | |  |  |
| Beidas 2012 | ***Checklist (range 0-6)*** | | | | | | | | | | | | | | | ***Checklist (range 1-7)*** | | | | | | | | | | | | NR | |
|  | **W+VC**  Pre: 1.6 (1.1)  Post: 2.9 (1.5) | | **OLT+VC**  Pre: 1.7 (1.3)  Post: 2.7 (1.4) | | | | | | | | | | **W^+^+VC**  Pre: 1.5 (1.2)  Post: 3.4 (1.2) | | | **W+VC**  Pre: 3.0 (1.1)  Post: 4.1 (1.5) | | **OLT+VC**  Pre: 2.9 (1.3)  Post: 3.7 (1.5) | | | | | **W^+^+VC**  Pre: 2.7 (1.2)  Post: 3.7 (1.5) | | | | |  |  |
|  | Participant adherence significantly improved pre- to post-training; no significant differences between conditions, although W^+^ improved significantly more than OLT. 38% trained to criterion at post-training and 61% after 3 months consultation post-training. | | | | | | | | | | | | | | | Participant competence significantly improved pre- to post-training; no significant differences between conditions. 65% trained to criterion at post-training and 85% after 3 months consultation post-training. | | | | | | | | | | | |  |  |
| Bennett-Levy 2012 | **OLT+SC**  Pre: 4.2 (1.6)  Post: 6.2 (1.4)  4-week follow-up: 6.6 (1.2) | | | | | | **OLT**  Pre: 4.7 (1.2)  Post: 6.4 (0.9)  4-week follow-up: 7.1 (1.2) | | | | | | | | |  | | | | | | | | | | | |  | |
|  | Both conditions significantly improved self-reported CBT skills from pre- to post-program and 4-week follow-up. No significant difference in skill acquisition between conditions at post-training or follow-up. | | | | | | | | | | | | | | |  |  |  |  |  |  |  |  |  |  |  |  |  |  |
| Chu 2017 | NR | | | | | | | | | | | | | | | NR | | | | | | | | | | | | NR | |
| Cohen 2016 | ***% of clients (N) receiving TF-CBT with adherence*** | | | | | | | | | | | | | | |  | | | | | | | | | | | |  | |
|  | **OLT+W+PhC**  *Initiated at least 1 session:*  55.6% (30/54)  *Completers:*  96% (25/26) | | | | | | | | **OLT+OC**  *Initiated at least 1 session:*  18.5 (5/27)  *Completers:*  83% (5/6) | | | | | | |  |  |  |  |  |  |  |  |  |  |  |  |  |  |
|  | Among initiators, therapists in OLT+W+C condition, reported significantly higher fidelity compared to OLT+OC. However, no differences were found for treatment completers. | | | | | | | | | | | | | | |  |  |  |  |  |  |  |  |  |  |  |  |  |  |
| Cooper 2017 | NR | | | | | | | | | | | | | | | NR | | | | | | | | | | | | NR | |
| Dimeff 2009 | ***PBRP (maximum score=9)*** | | | | | | | | | | | | | | | ***PBRP (range 0-5)*** | | | | | | | | | | | | NR | |
|  | **TM**  Pre: 8.0  (7.7)  Post: 20.8 (17.8)  90-day follow-up: 17.9 (14.7) | | **OLT**  Pre: 8.2  (6.5)  Post: 25.5 (12.1)  90-day follow-up: 22.8 (10.5) | | | | | | | | | | **W**  Pre: 8.5  (6.3)  Post: 23.1 (11.6)  90-day follow-up: 22.8 (14.8) | | | **TM**  Pre: 0.6 (0.8)  Post: 1.9 (1.31)  90-day follow-up: 1.4 (1.0) | | **OLT**  Pre: 0.6 (0.8)  Post: 1.9 (1.1)  90-day follow-up: 1.8 (0.9) | | | | | **W**  Pre: 0.8 (1.1)  Post: 2.1 (1.1)  90-day follow-up: 1.8 (0.9) | | | | |  |  |
|  | All conditions had significant improvement in adherence. There were no significant differences between conditions. | | | | | | | | | | | | | | | All conditions had significant improvement in competence. There were no significant differences between conditions in improvement in competence. At the 90-day follow-up, average competence scores indicated participants achieved minimal to moderate competency in applying DBT skills training. | | | | | | | | | | | |  |  |
| Dimeff 2011 | NR | | | | | | | | | | | | | | | NR | | | | | | | | | | | | NR | |
| Dimeff 2015 | NR | | | | | | | | | | | | | | | ***Global score (range 0-3)*** | | | | | | | | | | | | NR | |
|  |  |  |  |  |  |  |  |  |  |  |  |  |  |  |  | **OLT**  Pre: 0.8 (0.6)  90-day follow-up: 1.6 (0.8) | | **TM**  Pre: 0.8 (0.6)  90-day follow-up: 1.5 (0.8) | | | | | **W**  Pre: 0.9 (0.6)  90-day follow-up: 1.4 (0.6) | | | | |  |  |
|  |  |  |  |  |  |  |  |  |  |  |  |  |  |  |  | All conditions had significant increases in proficiency over time; no significant differences between conditions. Moderate proficiency achieved in all conditions at post-training and 90-day follow-up. | | | | | | | | | | | |  |  |
| Fu 2015 | ***MITI % MI adherent*** ***(range 0-100)*** | | | | | | | | | | | | | | | ***MITI Global Spirit (range 1-5)*** | | | | | | | | | | | | NR | |
|  | **W+BS+C**  Pre: 50% (37%)  3-month follow-up: 90% (19%) | | | | | | | | | **W**  Pre: 62% (38%)  3-month follow-up: 57% (39%) | | | | | | **W+BS+C**  Pre: 2.9 (0.6)  3-month follow-up: 3.2 (0.8) | | | | | **W**  Pre: 2.7 (0.8)  3-month follow-up: 2.5 (0.7) | | | | | | |  |  |
|  | W+BS+C had significantly greater improvement in MI adherence scores than W condition. | | | | | | | | | | | | | | | W+BS+C had significantly higher Global Spirit scores than the W condition. | | | | | | | | | | | |  |  |
| Gega 2007 | ***Skills total (range 0-8)*** | | | | | | | | | | | | | | | NR | | | | | | | | | | | | NR | |
|  | **OLT**  Post: 3.1 (1.2) | | | | | | | **W**  Post: 2.9 (1.5) | | | | | | | |  |  |  |  |  |  |  |  |  |  |  |  |  |  |
|  | Only assessed post-training. No significant differences were found between conditions post-training. | | | | | | | | | | | | | | |  |  |  |  |  |  |  |  |  |  |  |  |  |  |
| Harned 2011 | NR | | | | | | | | | | | | | | | NR | | | | | | | | | | | | NR | |
| Harned 2014 | NR | | | | | | | | | | | | | | | ***20-minute simulated role play, global score (range 1-5)*** | | | | | | | | | | | | NR | |
|  |  |  |  |  |  |  |  |  |  |  |  |  |  |  |  | **OLT**  Pre: 1.5 (0.1)  Post: 2.9 (0.1)  12-week follow-up: 2.8 (0.1) | | **OLT^+^**  Pre: 1.5 (0.1)  Post: 3.0 (0.1)  12-week follow-up: 3.0 (0.1) | | | | | **OLT^+^+LC**  Pre: 1.5 (0.1)  Post: 3.2 (0.1)  12-week follow-up: 3.2 (0.1) | | | | |  |  |
|  |  |  |  |  |  |  |  |  |  |  |  |  |  |  |  | All conditions had a significant increase in competence scores over time. OLT^+^+LC had significantly greater improvement in scores from baseline to 12-week follow-up than OLT. | | | | | | | | | | | |  |  |
| Henggeler 2008 | ***9-item CM cognitive behavioral adherence (range 1-4)*** | | | | | | | | | | | | | | | NR | | | | | | | | | | | | NR | |
|  | **W+IQA**  Raw data NR | | | | | | | | | **W**  Raw data NR | | | | | |  |  |  |  |  |  |  |  |  |  |  |  |  |  |
|  | Clients (youths) reported significant increase in therapist adherence to CM cognitive behavioral techniques in W+IQA condition but not in W condition. W+IQA scores were significantly higher than W at 4 months post-workshop. Youths’ caregivers reported an increase in therapist adherence in W+IAQ condition initially, but ratings returned to level close to baseline level at 4 months post-training. Ratings for therapists in the W condition initially decreased but returned to level close to baseline at 4 months post-training. | | | | | | | | | | | | | | |  |  |  |  |  |  |  |  |  |  |  |  |  |  |
|  | ***5-item CM monitoring adherence (range 1-3)*** | | | | | | | | | | | | | | |  |  |  |  |  |  |  |  |  |  |  |  |  |  |
|  | **W+IAQ**  Raw data NR | | | | | | | | | **W**  Raw data NR | | | | | |  |  |  |  |  |  |  |  |  |  |  |  |  |  |
|  | Clients (youths) and caregivers’ reports of therapist adherence for monitoring techniques showed no significant change from pre-training through 4-months post-training for either condition. | | | | | | | | | | | | | | |  |  |  |  |  |  |  |  |  |  |  |  |  |  |
| Hubley 2014 | NR | | | | | | | | | | | | | | | NR | | | | | | | | | | | | NR | |
| Larson 2013 | ***Mean Score (range 0 to 7)***  ***% “Low Pass” CBT adherence (≥4 on 7-point scale)*** | | | | | | | | | | | | | | | NR | | | | | | | | | | | | NR | |
|  | **OLT+S**  *Overall CBT skills*  Pre: 2.4; 17.0%  Post: 2.6; 28.3%  *Generic CBT skills*  Pre: 2.5; 45.3%  Post: 2.8; 49.1%  *CBT-specific skills*  Pre: 2.0; 26.4%  Post: 2.2; 34.0% | | | | | | | | | **TM+S**  *Overall CBT skills*  Pre: 2.3; 21.2%  Post: 2.8; 40.4%  *Generic CBT skills*  Pre: 2.3; 34.6%  Post: 2.8; 57.7%  *CBT-specific skills*  Pre: 2.2; 28.8%  Post: 2.4; 46.2% | | | | | |  |  |  |  |  |  |  |  |  |  |  |  |  |  |
|  | No significant gains in CBT skills pre- to post-training in either condition except for TM+S in generic CBT skills. There were no significant differences between conditions at post-training. The TM+S group had a significant increase in participants’ low pass rate from pre- to post-training in overall CBT and generic CBT skills. The conditions post-test low pass rates were not significantly different. | | | | | | | | | | | | | | |  |  |  |  |  |  |  |  |  |  |  |  |  |  |
| Martino 2011 | ***Adherence rating for fundamental MI Strategies (range 1-7) – client sessions*** | | | | | | | | | | | | | | | ***Competence rating for fundamental MI Strategies (range 1-7) – client sessions*** | | | | | | | | | | | | NR | |
|  | **TW+C**  Pre: 4.1 (1.0)  Post-workshop: 4.5 (0.9)  Post-supervision:  4.4 (1.1)  12-week follow-up: 4.4 (0.9) | | | | **EW+C**  Pre: 4.1 (0.9)  Post-workshop: 4.5 (0.8)  Post-supervision:  4.6 (0.8)  12-week follow-up:4.6 (0.8) | | | | | | | | | **TM^+^**  Pre: 3.7 (0.9)  Post-workshop: 3.7 (1.1)  Post-supervision:  4.0 (1.2)  12-week follow-up:3.9 (0.9) | | **TW+C**  Pre: 4.0 (0.7)  Post-workshop: 4.4 (0.9)  Post-supervision:  4.8 (0.7)  12-week follow-up: 4.4 (0.9) | | | **EW+C**  Pre: 4.1 (0.7)  Post-workshop: 4.8 (0.9)  Post-supervision: 4.6 (0.8)  12-week follow-up: 5.0 (0.9) | | | | | | **TM^+^**  Pre: 3.8 (0.8)  Post-workshop: 3.8 (0.9)  Post-supervision: 4.3 (0.9)  12-week follow-up: 3.9 (0.8) | | |  |  |
|  | ***Adherence rating for advanced MI Strategies (range 1-7) – client sessions*** | | | | | | | | | | | | | | | ***Competence rating for advanced MI Strategies (range 1-7) – client sessions*** | | | | | | | | | | | |  |  |
|  | **TW+C**  Pre: 2.5 (1.1)  Post-workshop: 2.6 (1.2)  Post-supervision:  2.9 (1.2)  12-week follow-up: 2.6 (1.1) | | | **EW+C**  Pre: 2.6 (0.8)  Post-workshop: 2.7 (0.9)  Post-supervision: 2.9 (0.9)  12-week follow-up: 2.7 (0.8) | | | | | | | | **TM^+^**  Pre: 2.3 (0.8)  Post-workshop: 2.5 (1.1)  Post-supervision: 2.7 (1.0)  12-week follow-up:  2.2 (0.7) | | | | **TW+C**  Pre: 4.0 (0.9)  Post-workshop: 4.2 (0.7)  Post-supervision: 4.6 (0.6)  12-week follow-up: 4.3 (0.7) | | | **EW+C**  Pre: 4.1 (0.7)  Post-workshop: 4.4 (0.8)  Post-supervision: 4.5 (0.6)  12-week follow-up: 4.9 (0.7) | | | | | | **TM^+^**  Pre: 4.1 (0.8)  Post-workshop: 3.9 (.9)  Post-supervision: 4.3 (0.9)  12-week follow-up: 4.0 (0.7) | | |  |  |
|  | In client sessions, no significant differences in adherence ratings between TW+C and EW+C. | | | | | | | | | | | | | | | In client sessions, EW+C had significantly greater increases in fundamental MI competence from pre- to post-workshop and 12-week follow-up and advanced competence from pre to 12-week follow-up compared to TM^+^. No significant differences in competence ratings between TW+C and EW+C in client sessions.  A significantly higher percentage of TW+C and EW+C participants achieved adequate MI performance standard post-supervision and 12-week follow-up compared to TM^+^ participants. | | | | | | | | | | | |  |  |
| McDonough 2002 | NR | | | | | | | | | | | | | | | NR | | | | | | | | | | | | NR | |
| Miller 2004 | ***MISC % MI-consistent responses*** | | | | | | | | | | | | | | | ***MISC Global MI Spirit (range 0-7), ≥ 5 indicates clinical proficiency*** | | | | | | | | | | | | NR | |
|  | **W**  Pre: 83.5 (18.6)  Post: 93.8 (12.5)  4-month follow-up: 90.5 (16.1) | **W+F**  Pre: 91.1 (11.3)  Post: 95.6 (7.6)  4- month follow-up: 98.3 (2.5) | | | | **W+C**  Pre: 86.8 (15.1)  Post: 96.3 (7.2)  4- month follow-up: 96.1 (8.1) | | | | | **W+F+C**  Pre: 92.9 (8.0)  Post: 97.8 (4.7)  4- month follow-up: 99.2 (1.9) | | | | **TM^+^**  Pre: 89.3 (11.4)  Post:  97.9 (3.3)*  4- month follow-up: 88.4 (16.7)* | **W**  Pre: 3.3 (1.5)  Post: 4.6 (1.6)  4- month follow-up: 4.1 (1.7) | **W+F**  Pre: 3.8 (1.3)  Post: 5.0 (1.3)  4- month follow-up: 5.1 (1.1) | | | | | **W+C**  Pre: 3.8 (1.6)  Post: 4.8 (1.2)  4- month follow-up: 5.0 (1.2) | | **W+F+C**  Pre: 3.7 (1.4)  Post: 5.0 (1.0)  4- month follow-up: 5.4 (0.9) | | | **TM^+^**  Pre: 3.7 (1.3)  Post: 4.9 (1.2)*  4- month follow-up: 4.1 (1.4)* |  | |
|  | All conditions had a significant increase in MI-consistent responses from pre- to post-workshop which was maintained at the 4-month follow-up. The TM**^+^** condition did not have a significant change in MI-consistent responses from baseline to 4-month follow-up and W, W+F, W+C, and W+F+C all had significantly greater gains in adherence at 4-months. There were no significant differences between W, W+F, W+C, and W+F+C conditions but only W+F, W+C, and W+F+C (not W or TM^+^) met 95% MI-consistent proficiency standard at 4- and 8-month follow-ups.  *TM**^+^** group completed workshop and post-workshop assessment after 4-month follow-up. | | | | | | | | | | | | | | | All conditions had significant increase in global MI spirit pre- to post-workshop which was maintained at the 4-month follow-up. The TM**^+^** condition did not have a significant change in spirit from baseline to 4-month follow-up W, W+F, W+C, and W+F+C all had significantly greater gains in spirit at 4-months. There were no significant differences between W, W+F, W+C, and W+F+C conditions but only W+F, W+C, and W+F+C (not W or TM^+^) met global MI spirit standard at 4- and 8-month follow-ups.  *TM**^+^** group completed workshop and post-workshop assessment after 4-month follow-up. | | | | | | | | | | | |  | |
| Monson 2018 | NR | | | | | | | | | | | | | | | NR | | | | | | | | | | | | NR | |
| Moyers 2008 | ***# of MI-adherent behaviors (M)*** | | | | | | | | | | | | | | | ***MITI Global MI Spirit, ≥5 indicates clinical proficiency*** | | | | | | | | | | | | NR | |
|  | **W**  Pre: 1.8 (2.1)  Post: 4.6 (3.1)  4-month follow-up: 1.4 (1.6) | | **W+F+C**  Pre: 2.0 (1.7)  Post: 4.9 (2.5)  4-month follow-up: 2.0 (1.7) | | | | | | | | | | **TM^+^**  Pre: 2.0 (2.4)  Post: 3.1 (2.3)  4-month follow-up: 1.9 (1.9) | | | **W**  Pre: 2.6 (0.8)  Post: 4.5 (1.1)  4-month follow-up: 3.9 (1.5) | | | | **W+F+C**  Pre: 3.1 (1.1)  Post: 4.8 (0.8)  4-month follow-up: 4.0 (1.4) | | | | | | **TM^+^**  Pre: 3.2 (1.2)  Post: 5.3 (0.9)  4-month follow-up: 3.0 (1.1) | |  |  |
|  | All conditions had a significant increase in MI-adherent behaviors from pre-to post-training. There were no significant differences between conditions at the 4-month follow-up. There was no significant improvement between pre-training and 4-month follow-up in frequency of MI-adherent behaviors. | | | | | | | | | | | | | | |  |  |  |  |  |  |  |  |  |  |  |  |  |  |
|  |  |  |  |  |  |  |  |  |  |  |  |  |  |  |  | All conditions had significant improvement in MI competence pre-to post-training. At 4-month follow up, both W and W+F+C conditions had significantly greater MI spirit than the TM^+^ condition. There was no significant difference between W and W+F+C conditions at the 4-month follow-up. | | | | | | | | | | | |  |  |
| Puspitasari 2017 | NR | | | | | | | | | | | | | | | ***28-item BASA (range 0-120)*** | | | | | | | | | | | | NR | |
|  |  |  |  |  |  |  |  |  |  |  |  |  |  |  |  | **DW**  Raw scores NR | | | | | **OLT**  Raw scores NR | | | | | | |  |  |
|  |  |  |  |  |  |  |  |  |  |  |  |  |  |  |  | Both conditions competency scores significantly increased from pre- to post-training and pre- to 3-month follow-up. The DW condition had significantly higher scores at post-treatment and follow-up than OLT. | | | | | | | | | | | |  |  |
| Rakovshik 2016 | NR | | | | | | | | | | | | | | | ***11-item CTS (range 0-6)*** | | | | | | | | | | | | NR | |
|  |  |  |  |  |  |  |  |  |  |  |  |  |  |  |  | **NT**  Pre: 2.1 (1.2)  Mid: 2.3 (1.3)  Post: 2.2 (1.2) | | **OLT+CW**  Pre: 2.4 (0.9)  Mid: 2.4 (1.0)  Post: 2.9 (1.0) | | | | | **OLT+CW+RS**  Pre: 2.3 (0.9)  Mid: 3.4 (0.8)  Post: 3.6 (0.9) | | | | |  |  |
|  |  |  |  |  |  |  |  |  |  |  |  |  |  |  |  | OLT+CW+S had a significantly greater increase in competence scores pre-to post-training than OLT+CW and NT conditions. OLT+CW and NT were not significantly different at post-training. | | | | | | | | | | | |  |  |
| Rawson 2013 | ***55-item YACS (range 1-7)*** | | | | | | | | | | | | | | | ***55-item YACS (range 1-7)*** | | | | | | | | | | | | NR | |
|  | **W+C**  Raw scores NR | | **DW+TC**  Raw scores NR | | | | | | | | | | **TM^+^**  Raw scores NR | | | **W+C**  Raw scores NR | | **DW+TC**  Raw scores NR | | | | | **TM^+^**  Raw scores NR | | | | |  |  |
|  | From pre-training to 12-weeks post-training, the DW+TC and W+C conditions had significantly higher adherence than the TM^+^ condition. W+C and DW+TC did not significantly differ. From weeks 12 to 24, all conditions had a decline in skills, with the W+C and DW+TC conditions having a significantly greater decline than the TM^+^ condition. Over the entire study period, DW+TC had significantly higher adherence than W+C and both DW+TC and W+C had significantly higher adherence than TM^+^. | | | | | | | | | | | | | | | At 4-weeks post-training, all conditions had a significant increase in competence scores and conditions did not significantly differ. From 4-weeks to 12-weeks post-training, the TM**^+^** group had a significant decline in scores, the DW+TC group did not change significantly, and the W+C group had a significant increase in competence scores. From 12-weeks (end of supervision for DW+TC and W+C conditions) to 24-weeks post-training, all groups had a significant decline in competence scores. At 24 weeks, the W+C condition had significantly higher competence scores than the DL+TC and TM**^+^** conditions. | | | | | | | | | | | |  |  |
| Ruzek 2014 | ***Role play rating (range 0-2)*** | | | | | | | | | | | | | | | NR | | | | | | | | | | | | NR | |
|  | **NT**  ME Pre: 0.5 (0.3)  ME Post: 0.5(0.3)  GS Pre: 0.7 (0.4)  GS Post: 0.8 (0.4)  BTA Pre: 0.7 (0.4)  BTA Post: 0.7(0.4) | | **OLT**  ME Pre: 0.6 (0.3)  ME Post: 0.7 (0.3)  GS Pre: 0.8 (0.3)  GS Post: 0.9 (0.5)  BTA Pre: 0.6 (0.3)  BTA Post: 0.8 (0.4) | | | | | | | | | | **OLT+C**  ME Pre: 0.6 (0.3)  ME Post: 0.9 (0.4)  GS Pre: 0.8 (0.4)  GS Post: 1.0 (0.4)  BTA Pre: 0.7 (0.3)  BTA Post: 0.9 (0.4) | | |  |  |  |  |  |  |  |  |  |  |  |  |  |  |
|  | OLT and OLT+C significantly improved on ME and BTA adherence compared to NT. OLT+C had significantly greater improvement in ME adherence than OLT. | | | | | | | | | | | | | | |  |  |  |  |  |  |  |  |  |  |  |  |  |  |
| Smith 2012 | ***Proportion (%) participants meeting MITI MI adherent proficiency threshold*** | | | | | | | | | | | | | | | ***Proportion (%) participants meeting MITI Spirit and Empathy threshold (≥5“beginner”, ≥6 “competent”)*** | | | | | | | | | | | | NR | |
|  | **W**  33% (7/21) | | **W+F**  53% (9/17) | | | | | | | | | | **W+LS**  50% (8/16) | | | **W**  ≥5  30% (8/27)  ≥6  0% (0/27) | | **W+F**  ≥5  46% (11/24)  ≥6  13% (3/24) | | | | | **W+LS**  ≥5  50% (12/24)  ≥6  25% (6/24) | | | | |  |  |
|  | Participants in the W+LS condition had significantly greater % of MI adherent than W only. W and W+AR and W+AR and W+LS were not significantly different. There was no significant difference between conditions in proportion of participants meeting MI adherent proficiency threshold. | | | | | | | | | | | | | | |  |  |  |  |  |  |  |  |  |  |  |  |  |  |
|  |  |  |  |  |  |  |  |  |  |  |  |  |  |  |  | W+LS had significantly greater scores on both MI Spirit and Empathy ratings at week 8 and week 20 than W only. W+AR had significantly greater MI Spirit scores than W only. No significant differences between W+LS and W+AR. W+LS had a significantly higher proportion of participants meeting MITI spirit and empathy than W at the ≥6 competency threshold. | | | | | | | | | | | |  |  |
| Weingardt 2006 | NR | | | | | | | | | | | | | | | NR | | | | | | | | | | | | NR | |
| Zatzick 2014  Darnell 2016 | ***MITI % MI adherent behaviors*** | | | | | | | | | | | | | | | ***MITI Global MI Spirit (range 1-5)*** | | | | | | | | | | | |  | |
|  | **W+F+C**  Pre: 18.1% (27.7%)  Post: 75.0% (36.3%)  4-month follow-up:  90.4% (22.0%)  27-month follow-up:  81.8% (31.8%) | | | | | | | | | **NT**  Pre: 37.5% (41.1%)  Post: 35.4% (38.7%)  4-month follow-up: 35.3% (39.3%)  27-month follow-up:  34.9% (40.2%) | | | | | | **W+F+C**  Pre: 2.9 (0.8)  Post: 3.9 (0.4)  4-month follow-up:  4.1 (0.8)  27-month follow-up:  3.9 (0.6) | | | | | **NT**  Pre: 2.6 (0.9)  Post: 3.0 (0.8)  4-month follow-up: 2.5 (0.8)  27-month follow-up:  2.9 (0.9) | | | | | | |  |  |
|  | The W+F+C condition had significantly greater MI adherence than the NT condition. W+F+C had a significant increase in MI adherence from pre-training to 27-month follow-up; the NT condition did not. | | | | | | | | | | | | | | | The W+F+C condition had significantly greater MI competence than the NT condition. W+F+C had a significant increase in competence from pre-training to 27-month follow-up; the NT condition did not. | | | | | | | | | | | |  |  |

**Table 3b. Study Outcomes: Participant Satisfaction, EBP Treatment Knowledge, and EBP Adoption**

| **Study** | **Participant satisfaction w/ training**  ***Mean (SD)***** | | | | | | **Participant EBP treatment knowledge**  ***Mean (SD)***** | | | | **Participant EBP adoption**  ***Mean (SD)***** | | | |
| --- | --- | --- | --- | --- | --- | --- | --- | --- | --- | --- | --- | --- | --- | --- |
| Bearman 2017 | NR | | | | | | NR | | | | NR | | | |
| Beidas 2012 | ***Satisfaction questionnaire (range 12-60***) | | | | | | ***20-item knowledge test (range 0-20)*** | | | | NR | | | |
|  | **W**  Post: 53.7 (5.4) | | **OLT**  Post: 50.8 (5.9) | | **W^+^**  Post: 55.5 (4.7) | | **W**  Pre: 15.3 (2.3)  Post: 17.5 (1.8) | **OLT**  Pre: 14.9 (2.4)  Post: 17.4 (1.8) | | **W^+^**  Pre: 15.3 (1.9)  Post: 16.8 (1.9) |  |  |  |  |
|  | Participants in W+ and W were significantly more satisfied than those in OLT. | | | | | | Participant knowledge significantly improved pre- to post-training; no significant differences between conditions*.* | | | |  |  |  |  |
| Bennet-Levy 2012 | NR | | | | | | ***20 item CBT-Q (range 0-20)*** | | | | ***Self-repot of 27 skills used with last 5 clients (range 0-27)*** | | | |
|  |  |  |  |  |  |  | **OLT+**  Pre: 9.8 (2.9)  Post:12.9 (2.8)  4-week follow-up: 12.7 (2.5) | | **OLT**  Pre: 11.0 (2.7)  Post:13.4 (3.0)  4-week follow-up: 13.0 (2.9) | | **OLT+**  Pre: 12.8 (3.5)  Post:18. 0 (4.8)  4-week follow-up: 18.1 (6.8) | | **OLT**  Pre: 15.1 (3.9)  Post:16.6 (6.4)  4-week follow-up: 18.2 (6.9) | |
|  |  |  |  |  |  |  | ***27-item CBT-knowledge test (range NR)*** | | | | CBT was significantly greater in the OLT group at baseline. There were no significant differences at post-training or 4-week follow-up. | | | |
|  |  |  |  |  |  |  | **OLT+**  *CBT-Knowledge*  Pre: 4.5 (1.7)  Post: 6.9 (1.1)  4-week follow-up: 7.2 (1.0) | | **OLT**  *CBT-Knowledge*  Pre: 5.2 (1.30)  Post: 7.2 (1.0)  4-week follow-up: 7.6 (1.2) | |  |  |  |  |
|  |  |  |  |  |  |  | Both groups significantly improved knowledge from pre- to post- training and follow-up. No significant difference in treatment knowledge between groups at post-training or follow-up for either measure. | | | |  |  |  |  |
| Chu 2017 | ***7-item survey (range 1-7)*** | | | | | | ***20-22 item knowledge test (range 0-22)*** | | | | ***Self-reported use with clients (range 0-3)*** | | | |
|  | **OLT+FS**  Mid: 3.9 (0.7)  Post: 3.8 (0.8) | | **OLT+ES**  Mid: 3.5 (1.2)  Post: 3.5 (1.0) | | **W+PC**  Mid: 3.4 (1.3)  Post: 3.4 (1.2) | | **OLT+FS**  Pre: 16.9 (1.2)  Mid: 13.6 (2.4)  Post: 14.4 (2.7) | **OLT+ES**  Pre: 16.1 (2.2)  Mid: 14.5 (2.6)  Post: 13.7 (2.1) | | **OLT+PC**  Pre: 17.3 (1.7)  Mid: 14.3 (1.8)  Post: 14.0 (1.8) | **OLT+FS**  Pre: 1.1 (0.5)  Mid: 1.2 (0.7)  Post: 1.3 (0.9) | **OLT+ES**  Pre: 1.1 (0.4)  Mid: 0.8 (0.7)  Post: 1.0 (0.9) | | **OLT+PC**  Pre: 1.0 (0.6)  Mid: 1.4 (0.6)  Post: 1.5 (1.0) |
|  | There were no significant differences in satisfaction between conditions at post-program. | | | | | | Significant decrease in knowledge across all conditions pre- to post-training. There were no significant differences between conditions. | | | | Use of CBT strategies increased significantly in all conditions pre- to post-training. There were no significant differences between conditions. | | | |
| Cohen 2016 | NR | | | | | | NR | | | | NR | | | |
| Cooper 2017 | NR | | | | | | ***22-item knowledge test (range 0-22)*** | | | | NR | | | |
|  |  |  |  |  |  |  | **OLT**  *Mean (IQR)*  Pre: 6.6 (1-19)  Post: 11.6 (0-18)  6-month follow-up: 11.0 (3-18) | | **OLT+SC**  *Mean (IQR)*  Pre: 7.3 (2-16)  Post: 11.7 (4-19)  6-month follow-up: 11.2 (2-17) | |  |  |  |  |
|  |  |  |  |  |  |  | Significant increase in knowledge in both conditions pre- to post-training and pre- to follow-up. No significant difference between conditions. Just over half (51%) of participants scored above competency threshold. | | | |  |  |  |  |
| Dimeff, 2009 | ***14-item survey (range 1-5)*** | | | | | | ***82-item test (range 1-100), (% of items correct)*** | | | | ***Self-reported use of skill in past 30-days (range 0-6)*** | | | |
|  | **MT**  *Learning Obj.*  Post: 3.3 (0.7)  *Training Meth.*  Post: 2.7 (0.9) | | **OLT**  *Learning Obj.*  Post: 3.8 (0.7)  *Training Meth.*  Post: 3.8 (0.8) | | **W**  *Learning Obj.*  Post: 3.8 (0.7)  *Training Meth.*  Post: 4.0 (0.8) | | **MT**  Pre: 27% (6%)  Post: 53% (16%)  90-day follow-up: 50% (17%) | **OLT**  Pre: 27% (6%)  Post: 61% (15%)  90-day follow-up: 58% (14%) | | **W**  Pre: 30% (7%)  Post: 56% (14%)  90-day follow-up: 52% (15%) | **MT**  90-day follow-up: 1.9 (1.2) | **OLT**  90-day follow-up: 2.0 (1.1) | | **W**  90-day follow-up: 1.7 (1.0) |
|  | OLT and W had significantly higher satisfaction ratings than MT for both learning objectives and effectiveness of training method. OLT and W did not differ significantly for either of these outcomes. | | | | | | All conditions had significant improvement in knowledge scores over time. OLT had significantly greater improvement than W and MT from pre- to post and from pre- to 90-day follow-up. W and MT did not significantly differ in change from pre- to post or pre- to 90-day follow-up. | | | | No significant differences between conditions in the self-reported number of DBT skills applied/taught in past 30 days. | | | |
| Dimeff 2011 | ***11-item survey***  ***(acceptability range 1-5, usability range 1-7)*** | | | | | | ***DBT Distress Tolerance Skills Knowledge and Application Test (range 0-100 %)*** | | | | ***Self-reported use of skills since training*** | | | |
|  | **OLT**  *‘Acceptability’*  Post: 3.9 (0.6)  15-week follow-up: 3.6 (0.8)  *‘Usability’*  Post: 5.8 (0.9)  15-week follow-up: 5.4 (1.0) | | **MT**  ‘Acceptability’  Post: 3.5 (0.7)  15-week follow-up: 3.0 (0.8)  *‘Usability’*  Post: 5.1 (1.3)  15-week follow-up: 4.5 (1.3) | | **pOLT**  ‘Acceptability’  Post: 3.1 (0.8)  15-week follow-up: 2.5 (1.0)  *‘Usability’*  Post: 5.9 (1.0)  15-week follow-up: 5.0 (1.4) | | **OLT**  Baseline: 20% (8%)  Post: 68% (15%)  15-week follow-up: 58% (20%) | **MT**  Baseline: 20% (14%)  Post: 64% (18%)  15-week follow-up: 51% (20%) | | **pOLT**  Baseline: 19% (10%)  Post: 21% (12%)  15-week follow-up: 25% (15%) | **OLT**  2-week follow-up: 2.3 (3.4)  7-week follow-up: 1.4 (1.4)  11-week follow-up: 1.2 (1.1)  15-week follow-up: 1.6 (2.0) | **MT**  2-week follow-up: 1.8 (1.6)  7-week follow-up: 1.0 (1.3)  11-week follow-up: 1.0 (1.6)  15-week follow-up: 1.0 (1.2) | | **pOLT**  2-week follow-up: 0.6 (1.2)  7-week follow-up: 0.4 (1.1)  11-week follow-up: 0.4 (1.0)  15-week follow-up: 0.4 (1.1) |
|  |  |  |  |  |  |  | All conditions had significant increase in knowledge over time. Both OLT and MT had significantly higher scores than pOLT at post-training and 15-week follow-up. OLT had a significantly higher score than MT at the 15-week follow-up. | | | |  |  |  |  |
|  | Both OLT and MT were rated significantly more acceptable than pOLT at post and 15-week follow-up and OLT was rated as significantly more acceptable than MT at both time points. OLT and pOLT rated significantly more usable than MT at post and 15-week follow-up; no significant differences in usability ratings between OLT and pOLT at post or 15-week follow-up. | | | | | |  |  |  |  | OLT participants reported significantly more skills use than pOLT participants at each follow-up timepoint and more than MT participants at the 7-week follow-up. MT participants reported significantly more skills use than pOLT at the 2-week follow-up. | | | |
| Dimeff 2015 | ***9-item generic scale (range 1-5)*** | | | | | | ***55-item chain analysis and 41-item validation knowledge test (% questions correct)*** | | | | ***Self-reported use of items (range 1- 6)*** | | | |
|  | **OLT**  Post: 3.6 (0.8) | **MT**  Post: 3.4 (0.7) | | | | **W**  Post: 4.0 (0.6) | **OLT**  Pre: 38% (8%)  n=55  60-day follow-up: 64% (13%)  90-day follow-up: 60% (12%) | **MT**  Pre: 38% (9%)  n=62  60-day follow-up: 56% (13%)  90-day follow-up: 54% (14%) | | **W**  Pre: 37% (8%)  n=55  60-day follow-up: 58% (12%)  90-day follow-up: 53% (12%) | **OLT**  Pre: 2.4 (1.2) n=29  60-day follow-up: 2.5 (1.1)  90-day follow-up: 2.5 (1.3) | **MT**  Pre: 2.7 (1.4) n=55  60-day follow-up: 2.5 (1.3)  90-day follow-up: 2.5 (1.4) | | **W**  Pre: 2.2 (1.1) n=50  60-day follow-up: 3.0 (1.3)  90-day follow-up: 2.7 (1.3) |
|  | W participants rated training significantly more satisfactory than OLT and MT participants. | | | | | |  |  |  |  |  |  |  |  |
|  |  |  |  |  |  |  | All participants had significant increases in knowledge pre-to post-training. OLT had greater knowledge at post-training and 90-day follow-up than MT and ILT. | | | | Participants had significantly increased use of strategies pre- to post-training; no significant differences between conditions*.* | | | |
| Fu 2015 | NR | | | | | | ***MI knowledge test (range 0-100)*** | | | | NR | | | |
|  |  |  |  |  |  |  | **W+BS+C**  Pre: 78% (14%)  3-month follow-up: 96% (6%) | | **W**  Pre: 81% (13%)  3-month follow-up: 90% (13%) | |  |  |  |  |
|  |  |  |  |  |  |  | The W+BS+C group showed significantly greater improvement in MI knowledge. | | | |  |  |  |  |
| Gega 2007 | ***3-item survey (range 0-8)*** | | | | | | ***20-item knowledge test (range 0-80)*** | | | | NR | | | |
|  | **OLT**  Post: 5.8 (1.1) | | | **W**  Post: 5.9 (1.2) | | | **OLT**  Pre: 66.0 (11.0)  Post: 73 (11.0) | | **W**  Pre: 63.0 (12.0)  Post: 72.0 (10.0) | |  |  |  |  |
|  | No significant difference in treatment satisfaction between groups. | | | | | | Both conditions had significant increases in knowledge from pre- to post-training. No significant differences between conditions in in treatment knowledge was found. | | | |  |  |  |  |
| Harned 2011 | ***Acceptability: 7-item survey (range 0-5) and Usability: 5-item survey (0-7)*** | | | | | | ***21-item knowledge test, (% questions correct)*** | | | | ***Self-reported use of 4-items*** | | | |
|  | **pOLT**  *‘Acceptability’*  Post: 3.3 (0.9)  *‘Usability’*  NR | | **OLT**  *‘Acceptability’*  Post: 3.9 (0.6)  *‘Usability’*  NR | | **OLT+MI**  *‘Acceptability’*  Post: 4.2 (0.6)  *‘Usability’*  NR | | **pOLT**  Pre: 33% (11%)  Post: 31% (14%)  1-week follow-up: 34% (17%) | **OLT**  Pre: 29% (9%)  Post: 71% (22%)  1-week follow-up: 65% (22%) | | **OLT + MI**  Pre: 26% (11%)  Post: 74% (13%))  1-week follow-up: 67% (17%) | **pOLT**  Pre: 0.5 (0.9)  1-week follow-up: 0.5 (1.0) | **OLT**  Pre: 0.8 (1.2)  1-week follow-up: 0.9 (0.9) | | **OLT + MI**  Pre: 0.4 (0.6)  1-week follow-up: 0.6 (1.0) |
|  | OLT and OLT+MI were rated as significantly more acceptable than pOLT at post-training. OLT and OLT+MI acceptability ratings did not significantly differ. There was no difference between conditions on usability ratings. | | | | | |  |  |  |  |  |  |  |  |
|  |  |  |  |  |  |  | All conditions knowledge test scores significantly improved over time. OLT and OLT+MI had significantly higher scores than pOLT at post-training and 1-week follow-up. There was no difference between OLT and OLT+MI knowledge scores at post-training or 1-week follow-up. | | | | No significant change pre-training to 1-week follow-up and no difference between conditions on use of skills in clinical practice. | | | |
| Harned 2014 | ***16-item (OLT) (range 16-112) and 9-item (ME and LC) questionnaire (range 1-5)*** | | | | | | ***49-item knowledge test (% items correct)*** | | | | ***Self-reported use of 9 exposure procedures per anxiety disorder client*** | | | |
|  | **OLT**  OLT Post: 84.2 (15.2) | | **OLT^+^**  OLT Post: 82.7 (17.3)  ME Post: 2.9 (0.8) | | **OLT^+^+LC**  OLT Post: 87.6 (17.2)  ME Post: 3.3 (1.0) | | **OLT**  Pre: 29% (2%)  Post: 64% (2%)  6-week follow-up:  61% (2%)  12-week follow-up: 63% (2%) | **OLT^+^**  Pre: 28% (2%)  Post: 62% (2%)  6-week follow-up:  59% (2%)  12-week follow-up: 62% (2%) | | **OLT^+^+LC**  Pre: 29% (2%)  Post: 68% (2%)  6-week follow-up:  65% (2%)  12-week follow-up: 68% (2%) | **OLT**  Pre: 0.9 (0.5)  Post: 2.8 (0.6)  6-week follow-up: 4.3 (0.6)  12-week follow-up: 3.3 (0.6) | **OLT^+^**  Pre: 1.1 (0.5)  Post: 2.9 (0.5)  6-week follow-up: 3.5 (0.6)  12-week follow-up: 4.2 (0.6) | | **OLT^+^+LC**  Pre: 0.8 (0.5)  Post: 2.7 (0.6)  6-week follow-up: 4.1 (0.6)  12-week follow-up: 4.9 (0.6) |
|  | No significant differences in OLT satisfaction between conditions. Significantly greater satisfaction with the motivational enhancement component in OLT^+^+LC than OLT^+^ condition. | | | | | |  |  |  |  |  |  |  |  |
|  |  |  |  |  |  |  | All conditions had significant increase in knowledge scores over time. OLT^+^+LC resulted in significantly greater increases in knowledge than OLT and OLT^+^ at all time points. | | | | No significant differences between conditions. At 12-week follow-up, 87.5% of participants reported at least some use of exposure procedures. | | | |
| Henggeler 2008 | NR | | | | | | NR | | | | NR | | | |
| Hubley 2014 | NR | | | | | | ***13-item knowledge test (% free recall items correct)*** | | | | NR | | | |
|  |  |  |  |  |  |  | **OLT**  *‘Knowledge’*  Pre: 33% (16%)  Post: 51% (15%)  1-week follow-up: 51% (13%)  *‘Recall’*  Pre: 1.0 (1.4)  Post: 14.5 (5.5)  1-week follow-up: 10.1 (4.4) | | **pOLT**  *‘Knowledge’*  Pre: 25% (13%)  Post: 34% (15%)  1-week follow-up: 33% (16%)  *‘Recall’*  Pre: 0.8 (1.3)  Post: 0.8 (1.4)  1-week follow-up: 1.3 (1.9) | |  |  |  |  |
|  |  |  |  |  |  |  | All participants significantly improved in the knowledge and recall tests from baseline to post-training and 1-week follow-up. OLT participants significantly outperformed pOLT participants at post-assessment and 1-week follow-up in both knowledge and recall. | | | |  |  |  |  |
| Larson 2013 | NR | | | | | | NR | | | | NR | | | |
| Martino 2011 | NR | | | | | | NR | | | | NR | | | |
| McDonough 2002 | ***2-item survey (range 0-8)*** | | | | | | ***75-item knowledge test (% items correct)*** | | | | NR | | | |
|  | **L+OLT**  *‘Educational value’*  Post: 4.3 (1.9)  *‘Enjoyment’*  Post: 3.5 (2.0) | | | **L+T**  *‘Educational value’*  Post: 5.6 (1.5)  *‘Enjoyment’*  Post: 5.8 (1.3) | | | **L+OLT**  Pre: 30.6% (11.2%)  Post: 57.1% (13.5%) | | **L+T**  Pre: 31.5% (16.2%)  Post: 66.5% (14.6%) | |  |  |  |  |
|  |  |  |  |  |  |  | Both conditions had significant increases in knowledge pre-to post-training. No significant difference between conditions was found. Both conditions were below mean expert’ scores (76.0%) post-training. | | | |  |  |  |  |
|  | The L+T condition was rated as significantly more educational and enjoyable than L+OLT condition. | | | | | |  |  |  |  |  |  |  |  |
| Miller 2004 | NR | | | | | | NR | | | | NR | | | |
| Monson 2018 | NR | | | | | | NR | | | | NR | | | |
| Moyers 2008 | NR | | | | | | NR | | | | NR | | | |
| Puspitasari 2017 | ***6-item TSQ (range 6-30)*** | | | | | | NR | | | | ***% using strategies with ≥1 client in past week*** | | | |
|  | **DW**  Session 1: 25.8 (3.4)  Session 2: 24.7 (4.0)  Session 3: 24.7 (3.8)  Session 4: 25.6 (3.3) | | | **OLT**  Session 1: 25.3 (3.1)  Session 2: 25.5 (2.8) Session 3: 25.1 (2.9)  Session 4: 26.1 (3.2) | | |  |  |  |  | **DW**  Pre: 28%  Post: 67%  3-month follow-up: 68% | | **OLT**  Pre: 29%  Post: 71%  3-month follow-up: 71% | |
|  | There was no significant difference in satisfaction between DW and OLT conditions for any of the four training sessions. | | | | | |  |  |  |  | Both DW and OLT conditions had a significant increase in use of BA strategies from pre-training to post-training and pre-training to 3-month follow-up. There were no significant differences between conditions. | | | |
| Rakovshik 2016 | NR | | | | | | NR | | | | NR | | | |
| Rawson 2013 | NR | | | | | | ***28-item knowledge test (range 0-28)*** | | | | NR | | | |
|  |  |  |  |  |  |  | **W**  Raw scores NR | **W+DL**  Raw scores NR | | **MT**  Raw scores NR |  |  |  |  |
|  |  |  |  |  |  |  | The W condition had a significantly greater gain in knowledge scores from baseline to 12-week post-training compared to the MT condition. The W+DL condition did not significantly differ from the W or MT conditions. | | | |  |  |  |  |
| Ruzek 2014 | NR | | | | | | ***12-item knowledge test (range 0-12), (# correct responses)*** | | | | ***Self-reported use of 12 subskills in past 30 days (range 1-5)*** | | | |
|  |  |  |  |  |  |  | **NT**  Pre: 7.8 (1.9)  Post: 8.0 (2.1) | **OLT**  Pre: 7.6 (1.9)  Post: 8.9 (1.9) | | **OLT+C**  Pre: 8.0 (2.0)  Post: 9.7 (1.7) | **NT**  Pre: 3.5 (0.8)  Post: 3.7 (0.8) | **OLT**  Pre: 3.6 (0.7)  Post: 4.0 (0.7) | | **OLT+C**  Pre: 3.5 (0.7)  Post: 3.7 (0.8) |
|  |  |  |  |  |  |  | OLT and OLT+C had significantly greater increases in overall CBT knowledge and ME and BTA modules than NT. | | | | All groups reported greater use of skills pre-to post-training. No significant differences between conditions. | | | |
| Smith 2012 | NR | | | | | | NR | | | | NR | | | |
| Weingardt 2006 | NR | | | | | | ***17-item multiple choice knowledge test*** | | | | NR | | | |
|  |  |  |  |  |  |  | **OLT**  Raw data NR | **W**  Raw data NR | | **NT**  Raw data NR |  |  |  |  |
|  |  |  |  |  |  |  | Participants in the OLT and W conditions showed significant improvement in mean knowledge test scores compared to NT participants. There was no significant difference between the OLT and W conditions. Participants in the NT condition had no improvement on scores post-training. | | | |  |  |  |  |
| Zatzick 2014  Darnell 2016 | NR | | | | | | NR | | | | NR | | | |

**Table 3c. Study Outcomes: Client Clinical Outcomes and Costs of Training**

| **Study** | **Client clinical outcomes** | | | | **Costs of training** | | | |
| --- | --- | --- | --- | --- | --- | --- | --- | --- |
| Bearman 2017 | NR | | | | | NR | | |
| Beidas 2012 | NR | | | | | NR | | |
| Bennet-Levy 2012 | NR | | | | | Online training (PRAXIS CBT for Common Mental Health Problems): $440 ($200 discount) per participant for access for 12-weeks for research study plus an additional 8 months post-study. | | |
| Chu 2017 | NR | | | | | NR | | |
| Cohen 2016 | ***% Clients Completed Treatment***  OLT+OC: 22%; OLT+W+C: 48%  After controlling for therapist effect, clients were more likely to complete treatment from OLT+W+C therapists than OLT+OC therapists.  ***22-item UCLA-RI for PTSD (range 0-88)***  Pre: 51.5 (18.7); Post: 37.0 (16.8)  *Client outcomes reported for therapy completers only. Client scores were not reported/compared by therapist training condition.  ***13-item MFQ (range 0-26)***  Pre: 12.9 (7.4); Post: 8.5 (6.7)  *Client outcomes reported for therapy completers only. Client scores were not reported/compared by therapist training condition. Clients who completed TF-CBT experienced statistically and clinically significant improvement in PTSD and depressive symptoms from pre- to posttreatment. | | | | | NR | | |
| Cooper 2017 | NR | | | | | NR | | |
| Dimeff, 2009 | NR | | | | | NR | | |
| Dimeff 2011 | NR | | | | | NR | | |
| Dimeff 2015 | NR | | | | | NR | | |
| Fu 2015 | NR | | | | | NR | | |
| Gega 2007 | NR | | | | | NR | | |
| Harned 2011 | NR | | | | | NR | | |
| Harned 2014 | NR | | | | | NR | | |
| Henggeler 2008 | NR | | | | | NR | | |
| Hubley 2014 | NR | | | | | NR | | |
| Larson 2013 | NR | | | | | NR | | |
| Martino 2011 | NR | | | | | NR | | |
| McDonough 2002 | NR | | | | | NR | | |
| Miller 2004 | NR | | | | | NR | | |
| Monson 2018 | **PCL-IV Total Score (*M*)** | | | | | NR | | |
|  | **W**  Pre: 60.6 (11.1)  Post: 49.1 (21.1) | **W+C**  Pre: 61.0 (11.5)  Post: 42.3 (13.4) | | **W+C^+^**  Pre: 61.7 (10.9)  Post: 49.5 (18.5) | |  |  |  |
|  | Clients treated by therapists in the W+C condition had a significantly greater decrease in PTSD symptom scores than those with therapists in the W condition. Client PTSD scores were not significantly different between the W+C and W+C^+^ condition or W+C^+^ and W. | | | | |  |  |  |
| Moyers 2008 | NR | | | | | NR | | |
| Puspitasari 2017 | NR | | | | | NR | | |
| Rakovshik 2016 | NR | | | | | NR | | |
| Rawson 2013 | NR | | | | | **W+C**  Per participant  $1,485  Total cost  $72,791 | **DW+TC**  Per participant  $768  Total cost  $37,648 | **TM^+^**  Per participant  $145  Total cost  $6,522 |
| Ruzek 2014 | NR | | | | | NR | | |
| Smith 2012 | NR | | | | | NR | | |
| Weingardt 2006 | NR | | | | | NR | | |
| Zatzick 2014  Darnell 2016 | **AUDIT hazardous drinking cutoff (%)** | | | | | Intervention training costs (workshop with feedback and coaching): $4300 | | |
|  | **W+F+C**  Admission: 71.4%  6-month follow-up: 61.7%  12-month follow-up: 56.9% | | **NT**  Admission: 66.0%  6-month follow-up: 59.0%  12-month follow-up: 59.6% | | |  |  |  |
|  | **AUDIT score (M)** | | | | |  |  |  |
|  | **W+F+C**  Admission: 10.5  6-month follow-up: 8.8  12-month follow-up: 8.2 | | **NT**  Admission: 9.7  6-month follow-up: 8.5  12-month follow-up: 8.5 | | |  |  |  |
|  | Clients in both conditions had significant reductions in AUDIT scores over the course of the year after hospital admission. Clients at W+F+C sites had an 8% reduction in AUDIT hazardous drinking cutoffs compared to NT sites and significantly greater reductions in continuous AUDIT scores than those at NT sites. | | | | |  |  |  |

**Abbreviations**

- **Evidence-based Psychotherapies (EBP):** CBT=Cognitive Behavioral Therapy; DBT=Dialectical Behavior Therapy; MET=Motivational Enhancement Therapy; MI=Motivational Interviewing
- **Training Conditions:** BS = Booster sessions;C = Consultation/Coaching; C^+^ = Consultation with audio review; CW = Consultation Worksheet; ES = Expert streaming consultation; EW = In-person workshop by expert; F = Audio review and feedback; FS = Fact Sheet; IQA = Intensive quality assurance; L = In-person lecture; LC = Learning Community; LS = Live teleconferencing supervision; NT = No training; OC = Online consultation course; OLT = Online training; OLT^+^ = Enhanced online training (components varied by study); PhC = Phone consultation; PC = Peer coaching/consultation; pOLT = Placebo online training; RS = Remote supervision; S = Supervision; SAU = Supervision as usual; SC = Supportive calls; SUP^+^ = Enhanced supervision (components varied by study); T = In; person group tutorial; TC = Telephone coaching; TM = Treatment manual TM^+^ = Enhanced training materials condition (components varied by study); TW = In-person workshop by trainee; VC = Virtual Consultation; W= In-person workshop; W^+^ = In-person enhanced workshop (components varied by study)
- **Assessment Measures:** AUDIT=Alcohol Use Disorders Identification Test; CTS=Cognitive Therapy Scale; MISC=Motivational Interviewing Skill Code; MITI=Motivational Interviewing Treatment Integrity; MFQ=Mood and Feelings Questionnaire; PCL-IV=Posttraumatic Stress Disorder Checklist - Fourth Edition; UCLA-RI for PTSD=UCLA Reaction Index for Posttraumatic Stress Disorder
- **Other:** DSM-IV=Diagnostic and Statistical Manual of Mental Disorders; MH = Mental Health; Fourth Edition; NR=Not Reported
